# GM000287 is a conserved determinant of serum resistance and virulence in *Klebsiella pneumoniae*

**DOI:** 10.1371/journal.ppat.1014619

**Published:** 2026-09-24

**Authors:** Xiaomin Lin, Junjun Jia, Zilin Zhuang, Yixin Mo, Xiaoxin Pan, Kaining Zhong, Ping Shen, Quanjiang Ji, Kai Zhou, Liang Yang, Yonghong Xiao, Chunfu Yang

**Affiliations:** 1 School of Public Health and Emergency Management, School of Medicine, Southern University of Science and Technology, Shenzhen, China; 2 SUSTech Homeostatic Medicine Institute, School of Medicine, Southern University of Science and Technology, Shenzhen, China; 3 School of Medicine, Southern University of Science and Technology, Shenzhen, China; 4 State Key Laboratory for Diagnosis and Treatment of Infectious Diseases, National Clinical Research Center for Infectious Diseases, National Medical Center for Infectious Diseases, Collaborative Innovation Center for Diagnosis and Treatment of Infectious Diseases, The First Affiliated Hospital, Zhejiang University School of Medicine, Hangzhou, China; 5 School of Physical Science and Technology, Shanghai Tech University, Shanghai, China; 6 Guangdong Provincial Key Laboratory of Regional Immunity and Diseases, Department of Pathogen Biology, Shenzhen University Medical School, Shenzhen University, Shenzhen, China; 7 Shenzhen Key Laboratory of Respiratory Diseases, Shenzhen Clinical Research Center for Respiratory Disease, Shenzhen Institute of Respiratory Diseases, Shenzhen People's Hospital (The First Affiliated Hospital, Southern University of Science and Technology, The Second Clinical Medical College, Jinan University), Shenzhen, China; Shandong University, CHINA

## Abstract

*Klebsiella pneumoniae* (*Kp*) is an opportunistic Gram-negative pathogen responsible for severe community- and healthcare-associated infections. Resistance to complement-mediated killing is an important determinant of *Kp* survival in human serum, yet mechanisms beyond capsular polysaccharide-mediated protection remain incompletely understood. Here, using comparative proteomic approaches, we identified a previously uncharacterized protein, GM000287, as a conserved determinant of serum resistance and virulence in *Kp*. Human serum exposure induced expression of the bacterial type II secretion system (T2SS), and deletion of the essential T2SS components GspD/GspE increased susceptibility to human serum killing. Importantly, disruption of GspD/GspE attenuated virulence in a murine pneumonia model. Comparative secretome analysis identified GM000287 as a T2SS-associated envelope protein that contributes to serum survival. Loss of GM000287 increased C3b production and factor B deposition on the bacterial surface during human serum exposure, consistent with enhanced alternative pathway amplification. In a murine pneumonia model, deletion of GM000287 reduced bacterial persistence and dissemination and attenuated virulence. Given the minimal bactericidal activity of mouse serum against *Kp*, indicating an additional role for GM000287 during infection. Importantly, GM000287 was highly conserved among *Klebsiella* species and broadly distributed among members of the *Enterobacteriaceae* family, and its contribution to serum resistance was validated in genetically diverse *Kp* clinical isolates. Together, these findings identify GM000287 as a conserved determinant of human serum resistance and *in vivo* virulence and reveal distinct contributions of GM000287 to *K. pneumoniae* fitness across different host-associated environments.

## Author summary

*Klebsiella pneumoniae* (*Kp*) is a major human pathogen responsible for severe infections, including pneumonia and bloodstream infections. To establish infection, *Kp* must withstand multiple host antimicrobial pressures, including complement-mediated killing in human serum; however, mechanisms beyond the well-characterized capsule-mediated resistance remain incompletely understood. Here, we identified GM000287, a previously uncharacterized and highly conserved protein, as an important determinant of human serum resistance and in vivo virulence. Comparative proteomic analysis identified GM000287 as a T2SS-associated envelope protein. Functional studies showed that GM000287 was required for optimal survival in human serum. Loss of GM000287 increased C3b production and factor B deposition on the bacterial surface during human serum exposure, consistent with enhanced alternative pathway amplification. Separately, deletion of GM000287 markedly attenuated *Kp* in a murine pneumonia model, resulting in reduced bacterial persistence and dissemination. Importantly, GM000287 was broadly conserved among *Klebsiella* species and related members of the *Enterobacteriaceae* family, and its contribution to serum resistance is conserved across genetically diverse *Kp* isolates. Together, these findings reveal a previously unrecognized bacterial factor with distinct contributions to human serum resistance and bacterial fitness during *Kp* infection.

## Introduction

*Klebsiella pneumoniae* (*K. pneumoniae*, *Kp*) is a Gram-negative bacterium belonging to the *Enterobacteriaceae* family. *Kp* has been classed as a critical priority pathogen by the World Health Organization,posing a substantial global health burden [1]. *Kp* infections are estimated to have caused the loss of approximately 31 million disability-adjusted life years (DALYs) worldwide. Of particular concern is the emergence of carbapenem-resistant *K. pneumoniae* (CRKP), which was linked to 929,000 deaths in 2019 [1]. The KPC-2-producing sequence type (ST) 11 clone accounts for 60–70% of CRKP cases in China [2–4]. In contrast, the ST258 clone carrying KPC-2 or KPC-3 is predominant in North America, Latin America, and Europe [4–8]. A recent study revealed that a single mutation in *recC* resulted in a highly virulent and multidrug-resistant ST11 subclone [9], further elevating the public health threat posed by CRKP.

Bacterial serum resistance is a key virulence trait that enables evasion of complement-mediated bactericidal activity in human serum [10]. Serum resistance in *Kp* is mediated by multiple surface-associated determinants, including capsular polysaccharide, lipopolysaccharide, and outer membrane proteins. Hypervirulent *K. pneumoniae* strains frequently carry virulence-associated determinants such as *rmpA*/*rmpA2*, *iucA*, *iroB*, and *peg-344*, some of which are associated with hypermucoviscosity and enhanced resistance to host defenses [11]. *Kp* can sequester complement proteins and neutralize their bactericidal effect [12]. In addition, capsule polysaccharide switching can physically impede assembly of the membrane attack complex on the bacterial membrane [13,14]. *Kp* reduces the initial binding and activation of complement cascades through modification of lipopolysaccharide (LPS) structure [15]. Beyond these well-characterized mechanisms, accumulating evidence indicates that diverse Gram-negative bacteria exploit surface-exposed proteins and hijack host complement regulators to suppress complement activation and promote serum resistance. For example, the surface-exposed *Escherichia coli* protein Iss is implicated in mediating serum resistance. *Kingella kingae* KK02920 and *Acinetobacter baumannii* recruit human factor H to their surfaces, thereby promoting the inactivation of surface-bound C3b [16,17]. However, the contribution of envelope-associated proteins to complement resistance in *Kp* remains incompletely defined [18,19].

Type II secretion system (T2SS) is widespread among Gram-negative bacteria and plays important roles in bacterial survival and virulence. T2SS translocates virulence factors from the periplasm across the outer membrane to the extracellular milieu, such as *Vibrio cholerae*, enterotoxigenic and enterohaemorrhagic *Escherichia coli* (ETEC and EHEC, respectively), *Pseudomonas aeruginosa*, *Legionella pneumophila* and *Yersinia enterocolitica* [20]. In *Klebsiella oxytoca*, the lipoprotein Pullulanase (PulA) is translocated to the cell surface through T2SS [21] and contributes to *Kp* immune evasion [22]*. Acinetobacter baumannii* and *Legionella pneumophila* utilize the T2SS to resist serum-mediated killing [23,24]. Whether the *Kp* T2SS contributes to adaptation to serum-associated host stress, and whether proteins functionally associated with this system participate in serum resistance, remain poorly understood.

Here, we investigated how *Kp* adapted to serum-associated host stress and identified the T2SS as a bacterial pathway induced by human serum exposure. Comparative proteomic analysis of wild-type Kp38088 and a T2SS-deficient mutant identified GM000287, a previously uncharacterized and highly conserved protein associated with T2SS-dependent envelope localization. GM000287 promoted resistance to human complement-mediated killing and limited C3b production and factor B deposition on the bacterial surface. Importantly, GM000287 contributed to bacterial fitness and virulence in a murine pneumonia model. Together, these findings identify GM000287 as a conserved determinant of human serum resistance and *in vivo* fitness, revealing distinct contributions of this previously uncharacterized protein across host-associated environments.

## Results

### Kp38088 exhibits host species- and inoculum-dependent serum resistance

One characteristic of CRKP is its resistance to serum killing, which is linked to the composition of the bacterial outer membrane and contributes to metastatic infection [25]. To characterize the serum resistance phenotype of Kp38088, we examined bacterial survival following exposure to normal human serum (NHS). Survival of Kp38088 was assessed at the indicated time points following incubation with 25%, 50%, or 75% NHS. Heat-inactivated normal human serum (HI-NHS) was included as a negative control. Kp38088 was inoculated at an initial density of 1 × 10^4^ CFU (Fig 1A) or 1 × 10^6^ CFU (Fig 1B). Kp38088 displayed resistance to complement-mediated killing, with no significant decrease in bacterial viability observed during the first 2 h of 50% NHS incubation with 1 × 10^6^ CFU. Notably, when a higher bacterial inoculum was used, Kp38088 maintained survival in 25% and 50% NHS even after prolonged exposure (6–12 h), highlighting its serum resistance phenotype.

**Fig 1:**
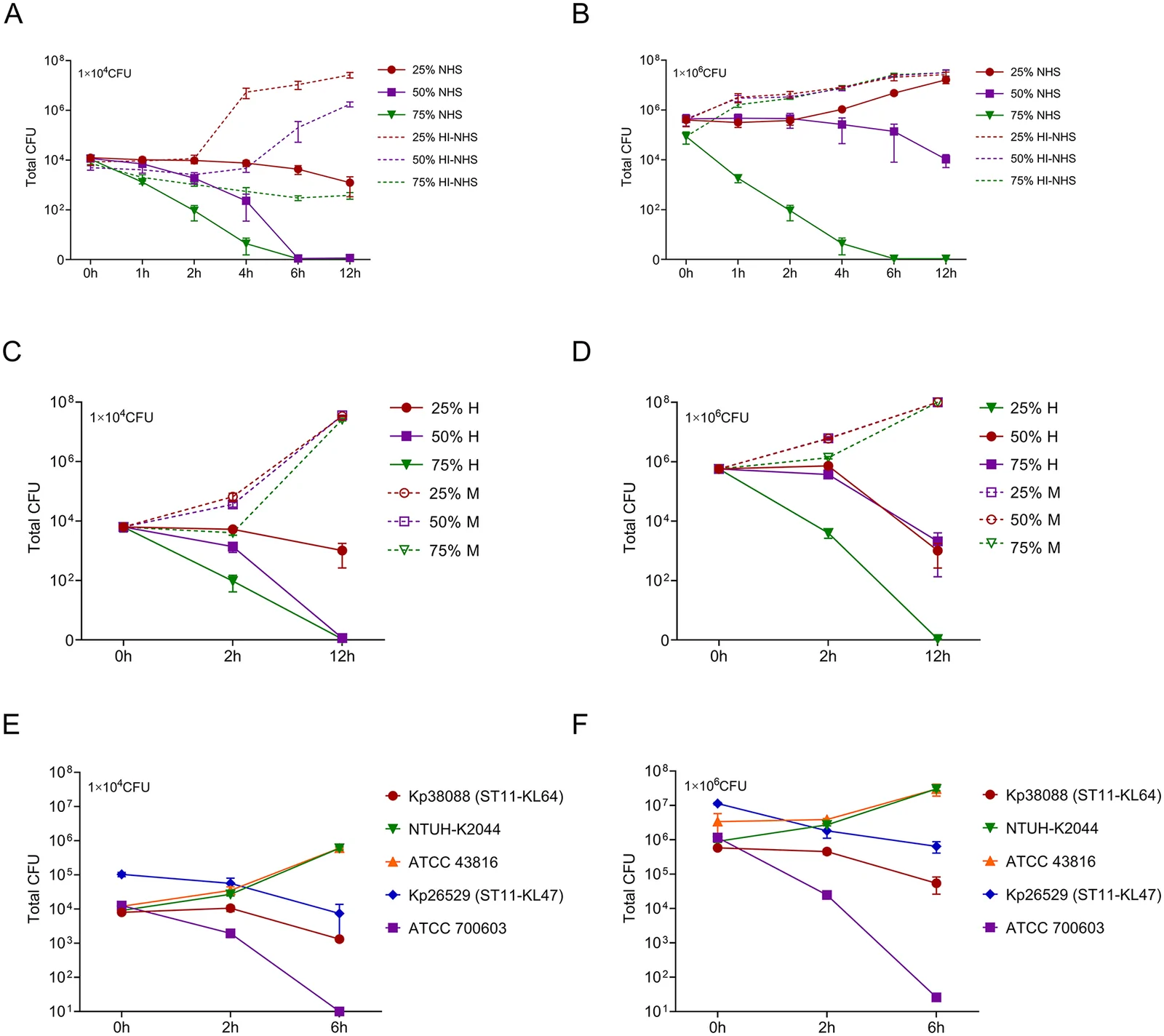
Kp38088 exhibits host species- and inoculum-dependent resistance to serum-mediated killin*g.* (A–B) Serum killing kinetics of Kp38088 in normal human serum. Kp38088 survival was monitored over time following incubation with 25%, 50%, or 75% NHS. Heat-inactivated serum was used as a negative control. Bacteria were inoculated at 1 × 10^4^ CFU (A) or 1 × 10^6^ CFU (B). Kp38088 exhibited resistance to complement-mediated killing at a higher inoculum, Kp38088 remained resistant to killing by 25% and 50% NHS after extended incubation (6–12 h), demonstrating a dose-dependent capacity to withstand serum-mediated antibacterial activity. (C–D) Species-dependent differences in serum-mediated killing of Kp38088. Kp38088 survival was assessed following incubation with normal sera from human serum (H) and C57BL/6 mouse serum (M), at the indicated concentrations. Bacterial survival was determined using an initial inoculum of 1 × 10^4^ CFU (C) or 1 × 10^6^ CFU (D). Mouse sera showed limited antibacterial activity against Kp38088 at both inoculum levels, whereas human serum exhibited substantial bactericidal activity. Notably, a high inoculum of Kp38088 displayed enhanced survival and resisted killing in 25% and 50% NHS. (E–F) Comparison of serum resistance among clinical *K. pneumoniae* isolates. Serum survival of different isolates, including ATCC 700603, Kp26529, Kp38088, NTUH-K2044, and ATCC 43816, was evaluated. Kp26529 and Kp38088 represent hypervirulent carbapenem-resistant *K. pneumoniae* (hv-CRKP) isolates, belonging to ST11-KL47 and ST11-KL64 lineages, respectively. Bacterial survival was determined in 25% NHS with an initial inoculum of 1 × 10^4^ CFU (E) and in 50% NHS with 1 × 10^6^ CFU respectively (F). Data were presented as the mean ± SEM of two independent experiments using five different normal human serum samples (A-B). Data were represented the means and SEM of three independent experiments (C-F).

To evaluate whether serum-mediated antibacterial activity differs among host species, Kp38088 was incubated with normal sera from humans (Fig 1C) and C57BL/6 mice (Fig 1D). Mouse serum exhibited minimal bactericidal activity against Kp38088 under the conditions tested, regardless of serum concentration or bacterial inoculum, which is consistent with a previous report [26]. In contrast, human serum exerted substantial bactericidal activity against Kp38088, with the extent of killing influenced by both serum concentration and bacterial burden. These findings demonstrate a marked host species-dependent difference in serum-mediated bactericidal activity.

We next assessed serum resistance across a panel of clinical *Kp* isolates with diverse genetic backgrounds. We compared the survival of five representative strains, including ATCC 700603, Kp26529, Kp38088, NTUH-K2044, and the well-studied *Kp* strain ATCC 43816. Under human serum exposure, these isolates exhibited heterogeneous survival profiles in NHS, indicating substantial strain-dependent differences in resistance to serum-mediated killing. Notably, the ST11 clinical isolates Kp26529 (ST11-KL47) and Kp38088 (ST11-KL64) displayed enhanced survival compared with the susceptible strain ATCC 700603 (CRKP) (Fig 1E-1F).

### T2SS contributes to Kp38088 resistance to human serum

The serum complement system serves as first line of host defense against bacterial infections. Resistance to serum-mediated killing increases the ability of *Kp* to establish infections. *Kp* adopts differing mechanisms and utilizes distinct gene sets to avoid complement attack [15]. To identify bacterial pathways responsive to human serum exposure, Kp38088 was incubated with 20% NHS or 1 × PBS for 1 h during exponential growth, followed by RNA sequencing. Using a fourfold change in transcript abundance between serum-treated and control samples as the threshold, RNA-seq analysis identified 92 upregulated and 51 downregulated genes following serum exposure (Fig 2A, S10 Appendix Dataset sheet 1). KEGG pathway analysis showed that the bacterial T2SS, along with metabolism and siderophore biosynthesis, exhibited the highest enrichment factors (Fig 2B). To validate these findings, qRT-PCR was performed on exponential-phase Kp38088 treated with 10%, 20%, and 30% human serum for one hour. The qRT-PCR results were consistent with the RNA-seq data, showing significant upregulation of T2SS genes *gspD* and *gspM* (Fig 2C).

**Fig 2:**
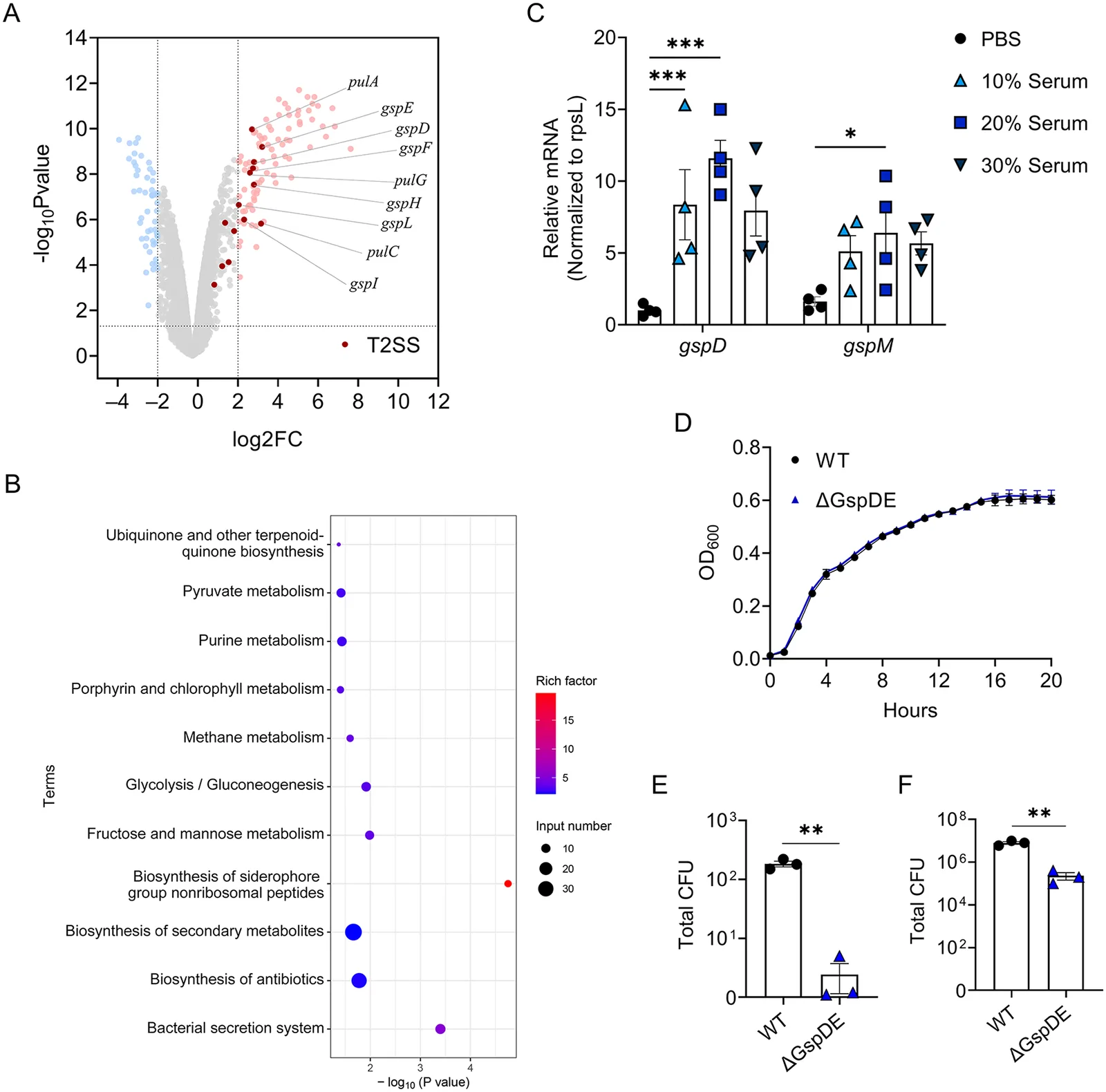
T2SS contributes to Kp38088 resistance to human serum-mediated killing. (A) A volcano plot of differentially expressed *Klebsiella pneumoniae* genes in response to serum treatment. T2SS-related genes highlighted as dark red dots were labeled. (B) KEGG pathway analysis was performed on the up-regulated genes following serum treatment. The y-axis represents the KEGG pathways, and the x-axis shows the -log10(P value). The size and color of the circles indicate the correlation of the up-regulated genes. (C) WT in the exponential stage were co-cultured with the indicated serum concentrations in LB medium for 1 hour. The expression of T2SS-related genes *gspD* and *gspM*, was quantified by qRT-PCR. Data are presented as the mean ± SEM from four independent experiments using normal human serum. (D) One step growth curve of WT and Δ*gspDE*. *Kp* strains were cultured in LB medium at a 1:100 inoculum ratio in 96-well plates, growth curves were recorded every 30 minutes over a 20-hour period. (E-F) 1 × 10^4^ (E) and 1 × 10^6^ CFU (F) of bacteria were resuspended in 1 × PBS and treated with 25% or 50% normal human serum respectively for 12 hours, CFUs of WT and Δ*gspDE* strains were enumerated. Data were represented the means and SEM of three independent experiments. Statistical significance was determined using unpaired two-tailed Student’s t- test; ***, P < 0.001; **, P < 0.01; *, P < 0.05; ns, not significant (2C, 2E, 2F).

Given that T2SS genes were up-regulated in response to serum *in vitro*, we hypothesized that T2SS might play a role in Kp38088 serum resistance. *Kp* T2SS contains a full complement of general secretory pathway (Gsp) proteins including GspABCDEFGH. GspD and GspE are essential components of T2SS [20]. To test the hypothesis, we first generated a Kp Δ*gspDE* mutant. Deletion of *gspDE* did not affect bacterial growth *in vitro* (Fig 2D). We then cocultured 1 × 10^4^ CFU of exponential-phase Kp38088 (termed wild-type (WT)) and Δ*gspDE* strains with 25% human serum (Fig 2E) or 1 × 10^6^ CFU of bacteria with 50% serum (Fig 2F) for 12 hours. The Δ*gspDE* strain exhibited significantly impaired survival compared with the WT strain. Together, these findings indicate that the Kp38088 T2SS contributes to resistance against human serum-mediated killing.

### T2SS promotes Kp38088 fitness and virulence during murine pulmonary infection

*Kp* T2SS is essential for its pathogenesis by exporting various factors, including Pullulanase (PulA) [27–29]. We first examined whether T2SS expression was induced during *in vivo* infection. Compared with bacteria grown in LB medium (*in vitro*), bacteria recovered from the lungs of WT-infected mice (*in vivo*) exhibited significantly increased transcription of the T2SS genes *gspD* and *gspM* (Fig 3A), suggesting that host-associated conditions promote T2SS activation during infection. We next utilized a non-invasive C57BL/6J mouse lung infection model to investigate the role of *Kp* T2SS in pathogenesis. The LD_50_ of WT Kp38088 following intratracheal infection of C57BL/6J mice was 3.3 × 10^6^ CFU per mouse (Fig 3B). The Δ*gspDE* mutant was significantly attenuated in the murine lung infection model, with a survival rate of 40% compared to 0% for the WT (Fig 3C). PulA has been reported as a virulence factor in *Kp* T2SS pathogenesis [22], we therefore tested a Δ*pulA* mutant in murine lung infection model, the Δ*pulA* mutant did not exhibit a significant difference in survival compared with the WT strain (S1 Fig). These findings indicate that the attenuation of the Δ*gspDE* mutant cannot be explained solely by loss of PulA and suggest that additional T2SS-associated factors contribute to Kp38088 fitness during pulmonary infection.

**Fig 3:**
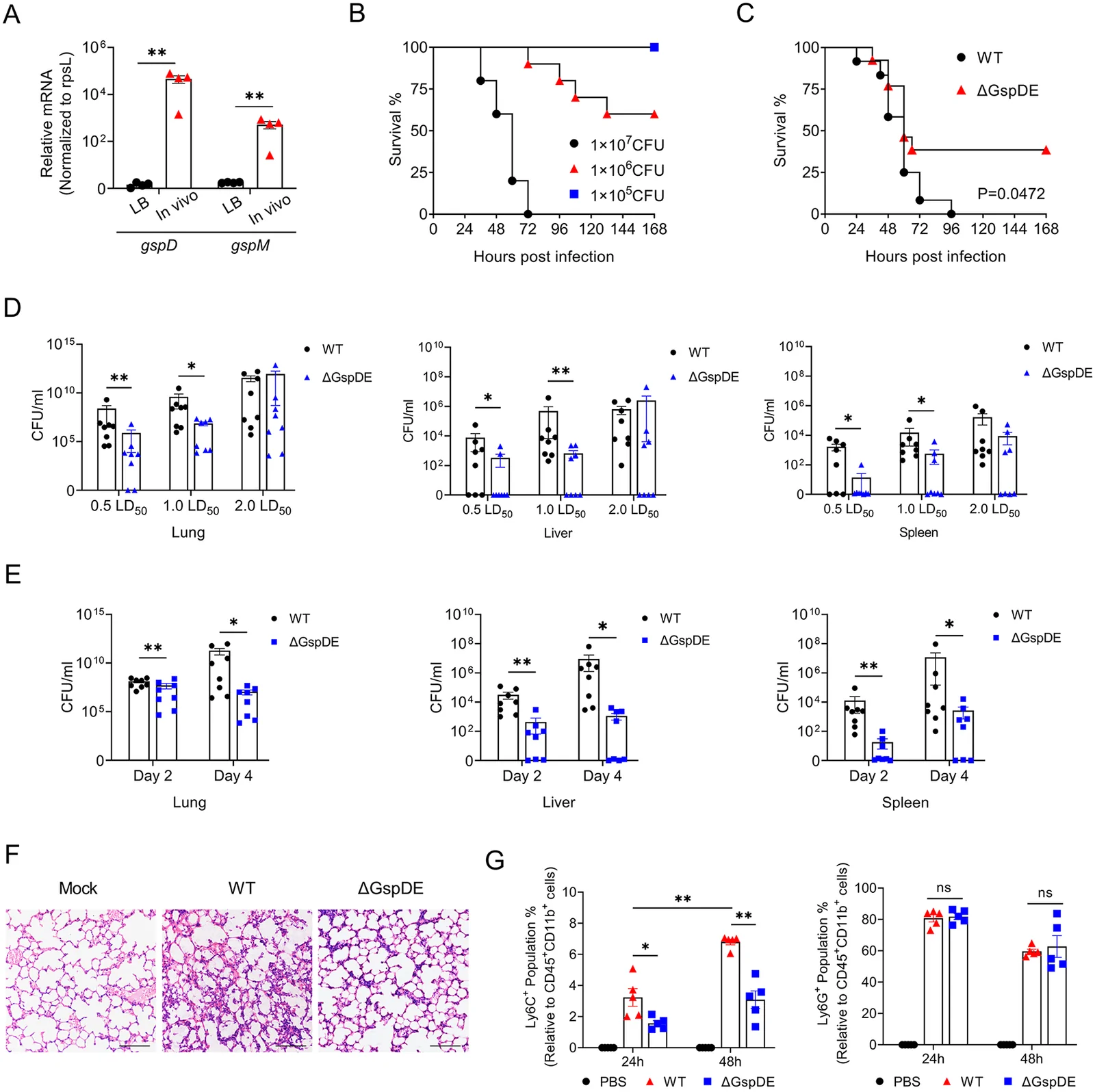
T2SS promotes Kp38088 fitness and virulence in mouse lung infection. (A) T2SS genes expression in WT-infected C57BL/6J mice. C57BL/6J mice were intratracheally infected with 2.0 LD_50_ WT strain, lung were harvest at 2 dpi. T2SS *gspD* and *gspE* gene were determined by qRT-PCR, WT grown in LB broth medium (*in vitro*) was used as the control for normalization. (B) LD_50_ of WT in mouse lung infection model. C57BL/6J mice were intratracheally infected with 1 × 10^5^, 1 × 10^6^, and 1 × 10^7^CFU respectively (n = 10). Survival was monitored for up to 7 dpi. LD_50_ was calculated by Kaplan–Meier method. (C) Survival curves of WT- and Δ*gspDE*-infected mice. C57BL/6J mice (n = 12) were intratracheally infected with 2.0 LD_50_ of WT or Δ*gspDE*. Survival was monitored for up to 7 dpi. (D) Bacterial burden in lung, liver and spleen from WT- and Δ*gspDE*-infected C57BL/6J mice. C57BL/6J mice were infected with 0.5, 1.0, or 2.0 LD_50_ of WT or Δ*gspDE* strains. Lung, spleen and liver tissues were harvested at 48 hpi and bacterial burden were determined by serial dilution and plating on agar. (E) Bacterial burden of lung, liver and spleen at 2 and 4 dpi. C57BL/6J mice were infected with 1.0 LD50 of WT or Δ*gspDE* strains. Lung, spleen and liver were harvested at 2 and 4 dpi, and bacterial burden was assessed by serial dilution and plating on agar. (F) Histopathological analysis of *Kp*-infected lung. Lungs from WT- and Δ*gspDE*-infected C57BL/6J mice (n = 3) were harvested at 2 dpi and subjected to hematoxylin and eosin (H&E) staining. (G) FACS Analysis of monocyte and neutrophil cells in WT- and Δ*gspDE*-infected BALF. Percentage of CD11b^+^Ly6C^+^ monocyte cell (L), percentage of CD11b^+^Ly6G^+^ neutrophil cell (M). Statistical significance was determined using unpaired two-tailed Mann-Whitney test. **, P < 0.01; *, P < 0.05; n.s., not significant (3A, 3D, 3E, 3G); Statistical significance of survival rate was determined using Mantel-cox test (3C).

We next assessed bacterial burden in the lung, liver and spleen of WT- and Δ*gspDE*- infected mice. Mice were infected with 0.5, 1.0, or 2.0 LD_50_ of WT or Δ*gspDE*, and organs were harvested on day 2 post-infection. Bacterial burden in lung, liver and spleen of the Δ*gspDE* mutant was significantly lower than that of the WT following infection with 0.5 LD_50_ and 1.0 LD_50_ infection (Fig 3D). We did not observe this difference when using 2.0 LD_50_ infection dose, suggesting that the contribution of T2SS to bacterial fitness is most readily detected at lower infection doses.

We also examined bacterial burden at both early and late stage of infection. C57BL/6J mice were intratracheally infected with 1.0 LD_50_ of WT and Δ*gspDE*. Bacterial burdens in lung, liver and spleen were monitored over time. The bacterial burden in lung, liver and spleen of Δ*gspDE* were significantly lower than WT-infected organs. By day 4 post-infection, bacterial burdens in the lungs, liver, and spleen of WT-infected mice had increased nearly 1,000-fold compared to day 2, whereas no significant changes were observed in the Δ*gspDE*-infected mice (Fig 3E). Histopathological analysis revealed that WT infection caused extensive leukocyte infiltration, primarily consisting of neutrophils and monocytes (Fig 3F). To determine whether the total number of neutrophils and monocytes in the bronchoalveolar lavage fluid (BALF) differed between WT and Δ*gspDE*-infected mice, we quantified CD11b^+^Ly6G^+^ neutrophils and CD11b^+^Ly6C^+^ monocytes by flow cytometry (S2A Fig). We found that the total number of CD45^+^CD11b^+^ cells was significantly higher in both WT and Δ*gspDE*-infected mice compared to mock-infected controls, but did not differ between the two infected groups (S2B Fig). Importantly, the number of CD11b^+^Ly6C^+^ monocytes in the BALF of Δ*gspDE*-infected mice was significantly lower than in WT-infected mice, whereas the number of CD11b^+^Ly6G^+^ neutrophils did not differ between the groups (Fig 3G). Together, these findings establish a contribution of T2SS to Kp38088 fitness and virulence during pulmonary infection.

### Identification and characterization of GM000287 as a determinant of serum resistance

To identify T2SS-associated proteins that could contribute to Kp38088 fitness under host-associated stress, we compared extracellular protein profiles of WT and Δ*gspDE* bacteria following envelope stress. Mass spectrometry identified 29 proteins that were exclusively detected in the extracellular fraction of the WT strain but absent in the Δ*gspDE* mutant (Fig 4A, S11 Appendix Dataset sheet 2). Functional annotation revealed that these proteins were involved in diverse biological processes, including envelope-associated functions (Fig 4B). Among these candidates, GM000287 was identified as a previously uncharacterized protein with an unknown function (Fig 4B, Table 1).

**Table 1:** The 29 proteins identified by mass spectrometry analysis of supernatants from WT and Δ*gspDE* strains. The supernatants from the WT and Δ*gspDE* strains were collected and analyzed by mass spectrometry. A total of 558 proteins were detected in the WT supernatant. Among these, 29 proteins were absent from the Δ*gspDE* supernatant (Table 1). Functional classification of the 29 proteins was conducted according to the COG database.

Accession	Annotation	COG	COG Class	SignalP-6.0 prediction
GM004341	transcriptional regulator BolA	KT	Transcription; Signal transduction mechanisms	
GM000669	peroxide/acid stress response protein YhcN	KT	Transcription; Signal transduction mechanisms	Sec/SPI
GM004371	6,7-dimethyl-8-ribityllumazine synthase	H	Coenzyme transport and metabolism	
GM001695	phospholipid-binding lipoprotein MlaA	M	Cell wall/membrane/envelope biogenesis	Sec/SPII
GM004529	2,5-didehydrogluconate reductase DkgB	Q	Secondary metabolites biosynthesis	
GM000287	hypothetical protein N559_0142	S	Function unknown	Sec/SPI
GM003751	leucine-responsive transcriptional regulator Lrp	K	Transcription	
GM001735	NUDIX hydrolase YfcD	I	Lipid transport and metabolism	
GM002106	pyruvate kinase	G	Carbohydrate transport and metabolism	
GM004575	iron-sulfur cluster insertion protein ErpA	O	Posttranslational modification, protein turnover, chaperones	
GM000543	phosphoglycolate phosphatase	C	Energy production and conversion	
GM000863	outer membrane channel protein TolC	M	Cell wall/membrane/envelope biogenesis	Sec/SPI
GM004761	2,3-diphosphoglycerate-dependent phosphoglycerate mutase GpmB	G	Carbohydrate transport and metabolism	
GM002301	YdiY family protein	M	Cell wall/membrane/envelope biogenesis	Sec/SPI
GM001601	Phosphoribosylformylglycinamidine cyclo-ligase, partial	F	Nucleotide transport and metabolism	
GM002130	RNA chaperone ProQ	T	Signal transduction mechanisms	
GM004746	molybdopterin adenylyltransferase	H	Coenzyme transport and metabolism	
GM003970	peptidoglycan-associated outer membrane lipoprotein	M	Cell wall/membrane/envelope biogenesis	Sec/SPII
GM000645	DNA-binding transcriptional regulator Fis	K	Transcription	
GM002337	iron ABC transporter periplasmic iron-binding protein	P	Inorganic ion transport and metabolism	
GM003568	N-acetylglucosamine kinase	R	General function prediction only	
GM003577	glycine zipper 2TM domain-containing protein	M	Cell wall/membrane/envelope biogenesis	# (27.7%)
GM002296	hypothetical protein AI2764V1_2077, partial	Q	Secondary metabolites biosynthesis	
GM004751	threonine synthase	E	Amino acid transport and metabolism	
GM000717	ribosome assembly RNA-binding protein YhbY	J	Translation, ribosomal structure and biogenesis	
GM004951	ribonuclease E inhibitor RraB	J	Translation, ribosomal structure and biogenesis	
GM002129	GAF domain-containing protein	T	Signal transduction mechanisms	
GM004262	co-chaperone YbbN	O	Posttranslational modification, protein turnover, chaperones	
GM000349	hypothetical protein	L	Replication, recombination and repair	

**Fig 4:**
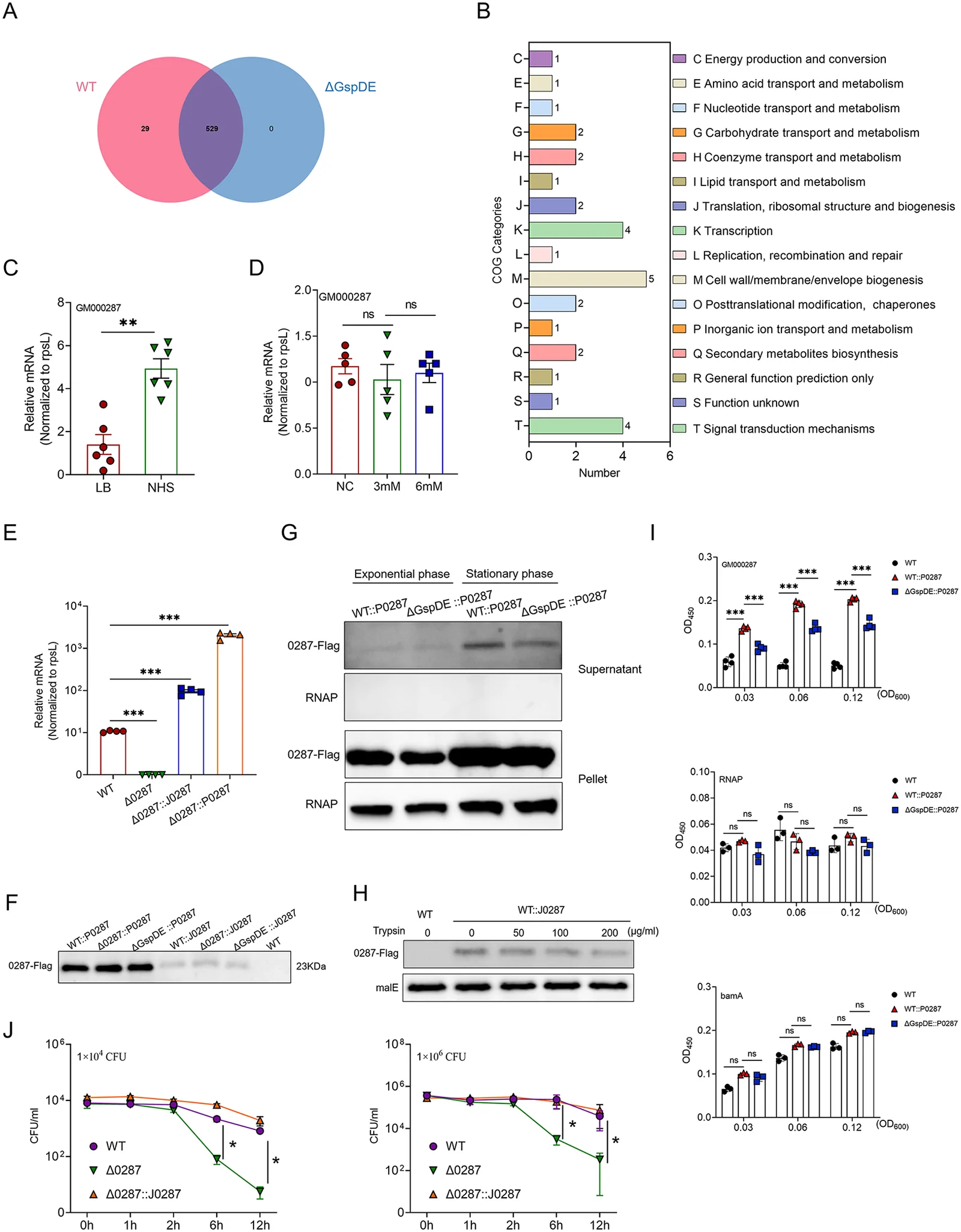
Identification and characterization of GM000287 as a T2SS-associated protein contributing to human serum resistance. (A–B) Identification of T2SS-dependent extracellular proteins. Extracellular proteins from WT and Δ*gspDE* Kp38088 strains following 3mM H_2_O_2_ exposure were analyzed by LC–MS/MS-based proteomics. A total of 29 proteins were uniquely detected in WT but absent from Δ*gspDE* supernatants, indicating their dependence on an intact T2SS for extracellular localization (A). These proteins were categorized according to Clusters of Orthologous Groups (COG) functional classification (B). (C–D) GM000287 expression in response to serum and oxidative stress. Kp38088 was exposed to 20% normal human serum (NHS) (C) or 3 mM H_2_O_2_ (D) for 1 h, and GM000287 transcript levels were quantified by qRT-PCR compared with 1 × PBS-treated controls. (E–F) Validation of GM000287 complementation. Δ0287 strains complemented with plasmids expressing GM000287 under either the native promoter (Δ0287*::*P0287) or J23103 promoter (Δ0287*::*J0287) were analyzed for GM000287 expression. Complementation restored GM000287 transcription (E) and protein production (F), as confirmed by qRT-PCR and immunoblotting using an anti-Flag antibody. (G) Accumulation of GM000287 in extracellular fractions. Extracellular GM000287 levels were monitored in WT*::*P0287 and Δ*gspDE::*P0287 strains during exponential and stationary growth phases by immunoblotting. RNA polymerase (RNAP) was used as a cellular contamination control. (H) Protease accessibility analysis of GM000287 surface exposure. Intact WT bacteria expressing Flag-tagged GM000287 were treated with increasing concentrations of trypsin for 30 min. Whole-cell proteins were subsequently analyzed by immunoblotting using an anti-Flag antibody. The periplasmic protein maltose-binding protein (MalE) was used as a protease-protected control. (I) Whole-cell ELISA detection of GM000287 localization in intact bacteria. Surface-associated GM000287 was detected in WT, WT*::*P0287, and Δ*gspDE::*P0287 strains using an anti-Flag antibody. BamA and RNA polymerase (RNAP) were detected as outer membrane and cytoplasmic controls, respectively, to validate membrane accessibility. (J) GM000287 contributes to serum resistance. Survival of WT, Δ0287, and complemented Δ0287*::*J0287 strains was assessed in 25% NHS with an initial inoculum of 1 × 10^4^ CFU and in 50% NHS with an initial inoculum of 1 × 10^6^ CFU respectively. Loss of GM000287 significantly impaired bacterial survival in human serum, whereas complementation restored serum resistance. Data were represented the means ± SEM from three independent experiments. Statistical significance was determined using unpaired two-tailed Student’s t-test. ***, P < 0.001; **, P < 0.01; *, P < 0.05; ns, not significant (4C, 4D, 4E, 4I and 4J).

Expression analysis revealed that GM000287 transcription was significantly induced following exposure to NHS, whereas its expression remained largely unchanged under H_2_O_2_-induced oxidative stress, suggesting that GM000287 is preferentially responsive to serum-associated stress conditions (Fig 4C–4D). To investigate the contribution of GM000287 to serum resistance, we generated a GM000287 deletion mutant (designated Δ0287) and two complemented strains in which GM000287 expression was driven either by its native 250-bp upstream regulatory region (Δ0287::P0287) or by the constitutive J23103 promoter coupled to the B0033 ribosome-binding site (Δ0287::J0287).

Both complementation constructs restored GM000287 expression, but to different levels (Fig 4E–4F). The J23103-driven construct (Δ0287::J0287) produced higher GM000287 transcript and protein levels than the wild-type strain, while remaining substantially lower than those observed in the native promoter-driven complemented strain (Δ0287::P0287). Despite successful restoration of GM000287 expression by both constructs, the two complemented strains exhibited distinct growth phenotypes. Δ0287 and Δ0287::J0287 showed growth kinetics comparable to those of the WT strain, whereas Δ0287::P0287, which expressed substantially higher levels of GM000287, displayed a measurable in vitro growth defect (S3A–S3C Fig).

We therefore primarily used Δ0287::J0287 for functional complementation assays, in which robust restoration of GM000287 expression allowed assessment of phenotype rescue, whereas Δ0287::P0287 was preferentially used for cellular and molecular analyses to minimize potential confounding effects associated with the growth defect observed upon high-level GM000287 expression.

To determine whether GM000287 localization was dependent on the T2SS, we first examined the transcriptional expression of GM000287 during the exponential and stationary growth phases and found no significant difference between the WT and Δ*gspDE* strains (S4 Fig), suggesting that deletion of *gspDE* does not affect GM000287 transcription. We next ectopically expressed GM000287 in both WT and Δ*gspDE* backgrounds and analyzed its subcellular distribution in WT*::*P0287 and Δ*gspDE::*P0287 strains by immunoblotting. Bacterial pellets and culture supernatants were collected and probed with an anti-Flag antibody to detect GM000287. RNA polymerase (RNAP) was used as a cytoplasmic protein control. GM000287 and RNAP were both detected in bacterial pellets from WT::P0287 and Δ*gspDE*::P0287 strains, whereas GM000287, but not RNAP, was detected in the culture supernatant (Fig 4G).

To further assess whether GM000287 was exposed on the bacterial surface, we performed protease accessibility assays. Treatment of intact bacteria with increasing concentrations of trypsin resulted in a dose-dependent reduction of Flag-tagged GM000287, whereas the periplasmic protein MalE remained protected under the same conditions (Fig 4H). We next examined whether GspDE/T2SS activity influences the surface accessibility of GM000287. Using whole-cell ELISA, we detected significantly reduced surface-associated GM000287 in the Δ*gspDE* mutant compared with the WT strain expressing Flag-tagged GM000287 (Fig 4I). Detection of BamA and RNAP served as controls for outer membrane and cytoplasmic localization, respectively, confirming the specificity of surface exposure analysis.

Given that GM000287 expression was induced upon serum exposure, we next examined its contribution to serum resistance. WT, Δ0287, and complemented Δ0287*::*J0287 strains were incubated with 25% NHS at an initial inoculum of 1 × 10^4^ CFU or with 50% NHS at an initial inoculum of 1 × 10^6^ CFU, and bacterial growth was monitored over 12 hours. The Δ0287 mutant exhibited a significantly impaired growth capacity compared with the WT strain under serum exposure, whereas complementation with J0287 restored growth to levels comparable to the WT (Fig 4J).

Together, these data support an envelope-associated and surface-accessible localization of GM000287 that is influenced by GspDE/T2SS activity and establish a role for GM000287 in human serum resistance.

### GM000287 is conserved across *Klebsiella* and contributes to human serum survival in diverse *Kp* strains

To determine the evolutionary conservation of GM000287 among *Klebsiella* species, we performed comparative genomic analysis of GM000287 homologs across 326 *Klebsiella* strains. GM000287 was highly conserved across the genus, with homologs detected in all analyzed *Klebsiella* species, including *K. pneumoniae* (269/269), *K. variicola* (16/16), *K. quasipneumoniae* (14/14), and other closely related species (Fig 5A, S12 Appendix Dataset sheet 3). Furthermore, a large-scale phylogenetic analysis of 1524 strains spanning major bacterial phyla revealed that GM000287 homologs are widely distributed with substantial representation within the *Enterobacteriaceae*. Furthermore, GM000287 homologs in multiple genera analysis of 143 representative strains from the *Enterobacteriaceae* family revealed that GM000287 homologs were broadly distributed among multiple genera, including *Escherichia*, *Salmonella*, *Serratia*, *Yersinia*, and *Shigella*, although the prevalence varied among species (Fig 5B). These findings indicate that GM000287 is an evolutionarily conserved protein within *Enterobacteriaceae*.

**Fig 5:**
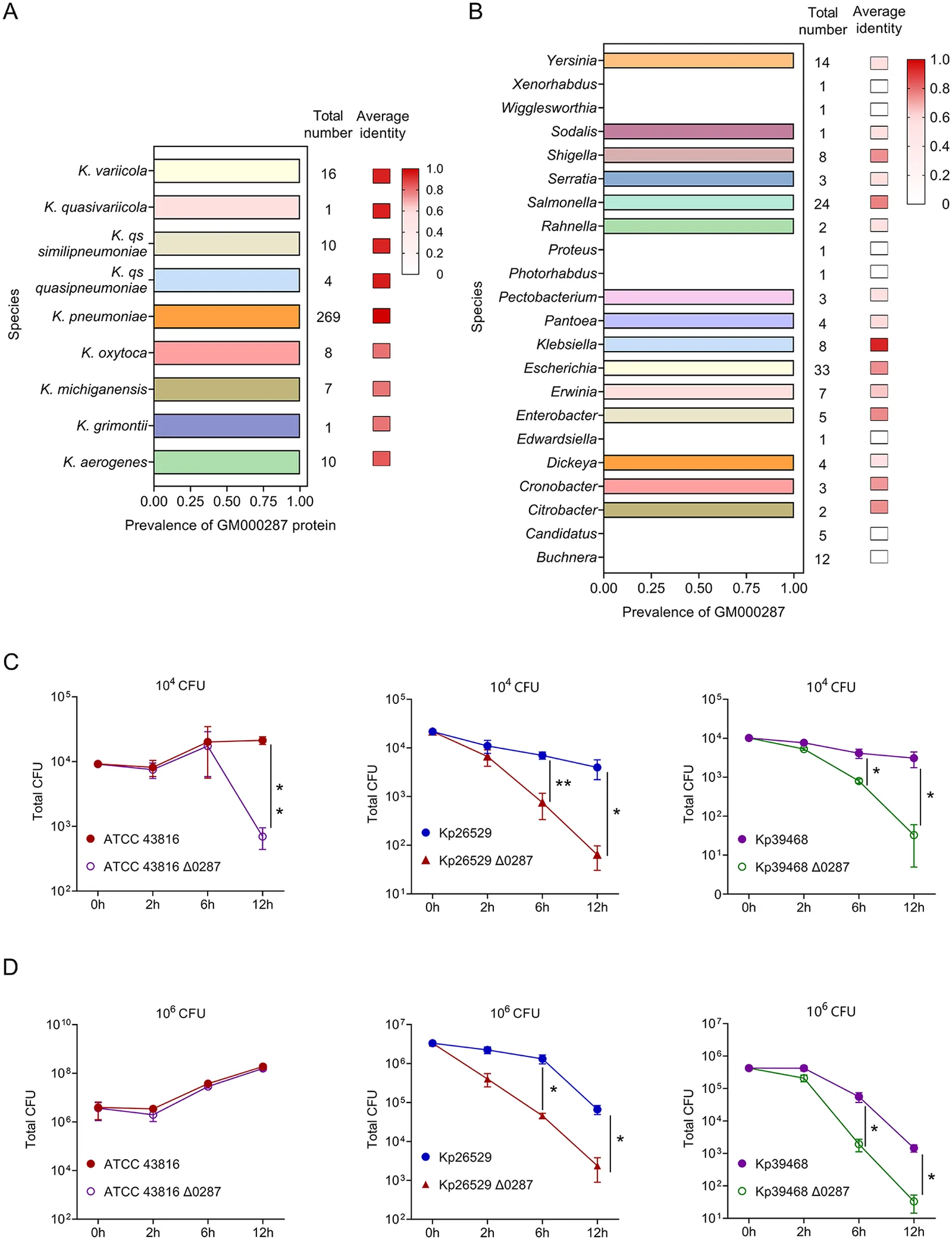
GM000287 is a conserved determinant of human serum resistance across diverse *K. pneumoniae* strains. (A–B) Conservation analysis of GM000287 among *Klebsiella* species and the *Enterobacteriaceae* family. The prevalence of GM000287 was calculated as the proportion of GM000287-positive strains within each species. Panel (A) included 326 Klebsiella strains, and panel (B) included 143 representativ*e Enterobacteriaceae* strains. The number of strains analyzed for each species was listed at the right of each bar. Average sequence identity represents the mean amino acid sequence identity among GM000287-positive strains within each species. (C–D) GM000287 contributes to serum resistance in multiple *Kp* strains. Wild-type strains (ATCC 43816, Kp26529, and Kp39468) and their corresponding ΔGM000287 mutants were incubated with NHS using an inoculum of 1 × 10^4^ CFU (C) or 1 × 10^6^ CFU (D), followed by CFU enumeration. Data were represented the means ± SEM from two independent experiments. Statistical significance was determined using unpaired two-tailed Student’s t-test. **, P < 0.01; *, P < 0.05; ns, not significant (5C and 5D).

T2SS was more widely distributed amon*g Gammaproteobacteria* (S5A Fig, S12 Appendix Dataset sheet 3). We further investigated the genetic organization and evolutionary conservation of the T2SS-associated genes identified in *K. pneumoniae*. The T2SS locus comprises a highly conserved cluster encoding the core secretion machinery components, including *gspC*, *gspD*, *gspE*, *gspF*, *gspG*, *gspH*, *gspI*, *gspJ*, *gspK*, *gspL*, and *gspM*, which are arranged within a contiguous genomic region. Notably, GM000287, a member of the YiiQ/DUF1454 family, was located outside the canonical T2SS gene cluster and was not genetically linked to the T2SS locus (S5B Fig, S12 Appendix Dataset sheet 3).

We next investigated whether GM000287 contributes to serum resistance in genetically distinct *Kp* strains. Clinical isolates including Kp26529, and Kp39468, and a highly used laboratory reference strain ATCC 43816, were selected for functional validation. Deletion of GM000287 significantly impaired bacterial survival during exposure to normal human serum in multiple independent *Kp* backgrounds. Deletion of GM000287 significantly increased serum susceptibility in all strains at the low inoculum. At the high inoculum, ΔGM000287 mutants also exhibited enhanced serum killing in all strains except ATCC 43816, whose survival was comparable to that of the corresponding wild-type strain (Fig 5C–5D). The defect in serum survival was most pronounced after prolonged serum exposure. Together, these data demonstrate that GM000287 is a highly conserved component among *Klebsiella* and *Enterobacteriaceae* and plays a role in serum resistance.

To determine whether GM000287 broadly influenced bacterial gene expression, we compared the transcriptomes of the Δ0287, Δ0287-Flag and WT strains. No significantly differentially expressed genes were identified in Δ0287 strain compared to WT (S6A Fig; S13 Appendix Dataset sheet 4), arguing against a major transcriptional regulatory role for GM000287 under the conditions tested. Δ0287-Flag showed defect growth curve compared to WT strain and expressed higher energy metabolic-related and membrane-associated genes (S6B Fig; S13 Appendix Dataset sheet 4), further indicated that GM000287 function as envelope-associated protein in serum resistance but not a genes expression regulator.

Additionally, it is possible that GM000287 anchors to the outer membrane, playing a role in bacterial adherence or internalization. To test this, adherence, internalization, and phagocytosis assays were performed in epithelial cell (MLE-12) and macrophage cell (BMDM). The Δ0287 strain showed no defects in adherence, internalization, or phagocytosis (S7A, S7B Fig). These findings indicate that loss of GM000287 does not measurably affect bacterial adherence, epithelial cell internalization, or macrophage uptake under the conditions tested.

### GM000287 contributes to bacterial fitness and virulence during murine pulmonary infection

Having demonstrated that GM000287 expression was significantly induced upon exposure to NHS *in vitro*, we next sought to determine whether GM000287 was similarly upregulated during pulmonary infection *in vivo*. BALF was collected from mice infected with the WT strain, and bacterial RNA was extracted to quantify GM000287 transcript levels. Consistent with the *in vitro* observations, GM000287 expression in bacteria recovered from infected lungs (*in vivo* group) was significantly elevated compared with bacteria cultured in LB medium (Fig 6A), suggesting that GM000287 is responsive to host-associated environmental cues during infection.

**Fig 6:**
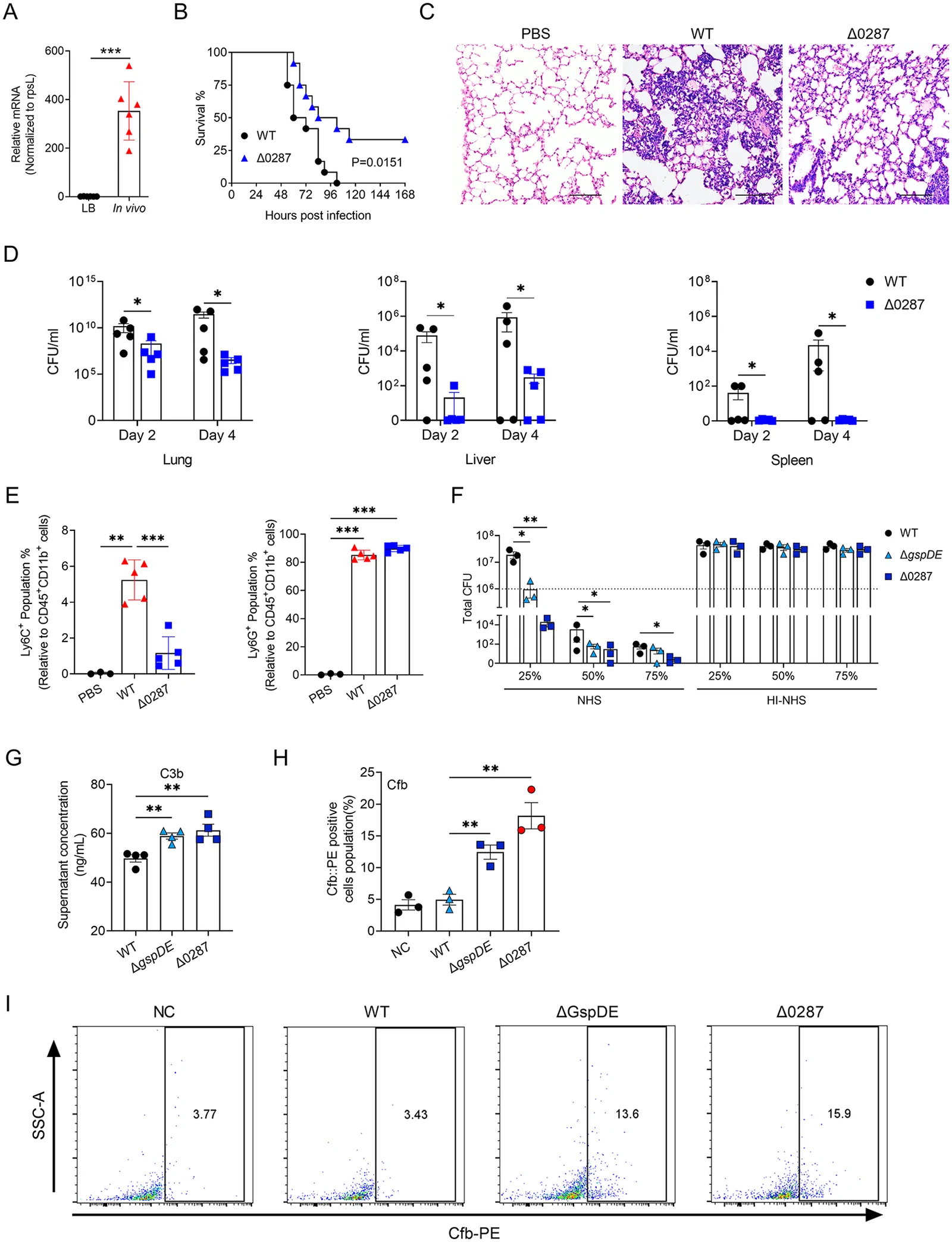
GM000287 contributes virulence in mice and resistance to human complement-mediated killing *in vitro.* (A) GM000287 expression in *Kp*-infected C57BL/6J mice. C57BL/6J mice were intratracheally infected with 2.0 LD_50_, lung were harvested at 2 dpi. GM000287 were determined by qRT-PCR, *Kp* grown in LB broth medium (*in vitro*) was used as control for normalization. (B) Survival rate of WT- and Δ0287- infected mice. C57BL/6J mice (n = 12) were i.t. infected with 2.0 LD_50_ WT and Δ0287 strains. Survival was monitored for up to 7 days. (C) Bacterial burdens of WT- and Δ0287-infected mice in lung, liver and spleen. C57BL/6J mice (n = 5) were infected with 1.0 LD_50_ WT and Δ0287 strains, lung, liver and spleen were harvested at 2 and 4 dpi, CFUs were enumerated. (D) Histopathological Analysis of *Kp*-infected lung. Lungs from WT- and Δ0287*-*infected C57BL/6J mice (n = 3) were harvested at 2 dpi and subjected to hematoxylin and eosin (H&E) staining. (E) FACS analysis of monocyte and neutrophil cells in WT- and Δ0287 -infected BALF. C57BL/6J mice (n = 5) were i.t. infected with 2.0 LD_50_ WT and Δ0287 strains. Percentage of CD11b+ Ly6C+ monocyte cell and CD11b+ Ly6G+ neutrophil cell was calculated. Infiltration of monocyte cells were significantly reduced in Δ0287-infected BALF at 48 hpi. (F) GM000287-mediated serum resistance is complement-dependent. 1 × 10^6^ CFU of WT, Δ*gspDE* and Δ0287 strains were co-cultured with varying concentrations of NHS or HI-NHS for 12 hours. CFUs were enumerated by plating serial dilutions. (G) Complement C3b levels in the supernatant following incubation of WT, Δ*gspDE*, and Δ0287 strains with normal human serum. (H-I) Flow cytometry analysis of Cfb deposition on bacteria surface. WT, ΔgspDE, and Δ0287 strains (3 × 10⁷ CFU) were co-cultured with varying concentrations of NHS for 45 minutes. Cfb levels were measured by flow cytometry. The percentage of Cfb in each sample was shown in (H), and representative flow cytometry plots were shown in (I). Data were represented the means and SEM of two independent experiments. Statistical significance was determined using unpaired two-tailed Student’s t- test (6A, 6E, 6H and 6I); Mann-Whitney U test (6F and 6G) ***, P < 0.001; **, P < 0.01; *, P < 0.05; ns, not significant; Statistical significance of survival rate was determined using Mantel-cox test (6B).

To investigate the contribution of GM000287 to *Kp* pathogenicity *in vivo*, mice were intratracheally infected with 2.0 LD_50_ of either the WT or Δ0287 strain. Compared with WT-infected mice, animals infected with the Δ0287 mutant exhibited significantly higher survival, indicating an attenuated virulence phenotype (Fig 6B). Consistently, bacterial loads in the lungs, liver, and spleen were significantly reduced in mice infected with the Δ0287 strain compared with those infected with the WT strain (Fig 6C). Histopathological analysis revealed that WT and Δ0287 infection caused extensive leukocyte infiltration, primarily consisting of neutrophil cells and monocytes (Fig 6D). To determine whether the total number of neutrophils and monocytes in the BALF differed between WT and Δ0287-infected mice, we quantified CD11b^+^Ly6G^+^ neutrophils and CD11b^+^Ly6C^+^ monocytes by flow cytometry. We found that the number of CD11b^+^Ly6C^+^ monocytes in the BALF of Δ0287-infected mice was significantly lower than in WT-infected mice, whereas the number of CD11b^+^Ly6G^+^ neutrophil cells did not differ between the groups (Fig 6E). Together, these findings demonstrate that GM000287 contributes to bacterial fitness and virulence during pulmonary infection.

### GM000287 contributes resistance to human complement-mediated killing

To determine whether the human serum susceptibility phenotype of the Δ0287 mutant depended on active complement, we compared bacterial survival in normal and heat-inactivated human serum. Consistent with our previous findings, incubation of WT and Δ0287 strains with 25%, 50%, or 75% NHS for 12 h revealed a substantial reduction in viable CFUs of the Δ0287 mutant compared with WT. In contrast, HI-NHS did not significantly affect the survival of Δ*gspDE* or Δ0287 mutants, with bacterial recovery comparable to WT (Fig 6F), indicating that the killing phenotype was dependent on complement activation.

To exclude the possibility that the contribution of GM000287 to serum resistance was indirectly mediated through alterations in capsule production, we quantified capsule levels in WT, Δ*gspDE*, Δ0287, and Δ*wcaJ* (S8 Fig). Neither deletion of the T2SS machinery nor loss of GM000287 significantly altered capsule production. These findings indicate that the serum susceptibility of the Δ0287 mutant is not attributable to altered capsule abundance.

Complement activation can proceed through the classical, lectin, or alternative pathways. To obtain initial insight into complement-associated responses during murine pulmonary infection, we examined complement-related gene expression in BALF. Transcripts encoding C3 and complement factor B (Cfb) were increased during *Kp* infection, whereas C1q, C2, and C4 were not significantly altered (S9 Fig). Because the serum susceptibility phenotype of the Δ0287 mutant depended on active complement, we next examined whether loss of GM000287 altered complement deposition during human serum exposure.

Incubation of WT and Δ0287 bacteria with NHS resulted in significantly increased C3b production in Δ0287 bacteria compared with WT bacteria (Fig 6G). Cfb (Bb fragment) deposition was significantly increased on both Δ*gspDE* and Δ0287 bacteria compared with WT bacteria (Fig 6H–6I). Together, these findings demonstrate increased C3b production and Cfb deposition in the absence of GM000287 during human serum exposure, a pattern consistent with enhanced alternative pathway amplification, and support a role for GM000287 in resistance to human complement-mediated killing.

## Discussion

*Klebsiella pneumoniae* employs diverse mechanisms and gene networks to evade complement-mediated killing [13,15,30–38]. The capsular polysaccharide (CPS) serves as a primary defense layer by restricting C3b deposition on the bacterial surface and reducing membrane attack complex (MAC) formation [39,40]. Similarly, O antigen contributes to serum resistance by interfering with MAC assembly and limiting macrophage activation [41]. Beyond these classical surface barriers, additional envelope factors have been implicated in complement evasion. The outer membrane porin OmpK36, for example, can interact with C1q to promote complement activation and subsequent C3b and C5b-9 deposition; paradoxically, loss of OmpK36 modestly enhances serum resistance while reducing bacterial virulence [42]. Terminal sialylation of CPS has also been shown to partially impair complement-mediated phagocytosis in hypermucoid *K. pneumoniae* strains [43]. Notably, highly virulent strains, including ATCC 43816 and RH201207, maintain serum resistance despite substantial C5b-9 deposition, suggesting that additional envelope-associated mechanisms contribute to functional resistance against MAC-mediated killing [19]. These observations suggest that serum resistance reflects the combined effects of structural and protein-associated determinants at the bacterial envelope.

*K. pneumoniae* encounters diverse envelope stresses, including complement attack and oxidative stress during infection, which require coordinated remodeling of the bacterial surface and maintenance of envelope integrity. Although the T2SS is classically recognized as a protein export machinery, emerging evidence suggests that it also participates in envelope homeostasis and adaptation to environmental stress [44,45]. In this study, human serum exposure induced expression of T2SS components, whereas deletion of *gspDE* markedly increased susceptibility to serum-mediated killing. These findings support a role for the T2SS in adaptation to serum-associated envelope stress. Consistent with this interpretation, comparative analysis of extracellular proteins from WT Kp38088 and the Δ*gspDE* mutant identified GM000287 as a T2SS-associated envelope protein required for optimal serum resistance. Importantly, however, the GspDE-dependent extracellular recovery of GM000287 did not establish GM000287 as a bona fide T2SS substrate. Rather, loss of GspDE may alter envelope organization, protein accessibility, localization, or release under stress conditions. Thus, our data established a functional relationship between the T2SS and GM000287 localization while leaving the precise nature of this relationship unresolved.

We next sought to define how GM000287 contributes to serum resistance. Complement activation proceeds through the classical, lectin, and alternative pathways, all of which converge on C3 activation and ultimately MAC formation. In addition to their roles in bacterial killing, complement-derived mediators such as C3a and C5a regulate leukocyte recruitment and activation [46–51]. During human serum exposure, deletion of either *gspDE* or GM000287 increased C3b production and factor B deposition on the bacterial surface, consistent with enhanced alternative pathway amplification. Moreover, the serum susceptibility phenotype of the ΔGM000287 mutant was abolished following heat inactivation, establishing that this phenotype depends on active complement. Together, these findings place GM000287 at the interface between the bacterial envelope and complement deposition. However, they do not define the molecular mechanism by which GM000287 alters complement activity. In particular, whether GM000287 directly interacts with a complement component or instead modifies the bacterial surface in a manner that secondarily affects complement deposition remains unknown. Identifying the bacterial or host interaction partners of GM000287 will therefore be important for resolving this mechanism.

A distinct question raised by our findings is how GM000287 contributes to virulence *in vivo*. GM000287 strongly promoted resistance to human serum, whereas this phenotype was not evident in mouse serum *in vitro*. Nevertheless, deletion of GM000287 markedly reduced bacterial persistence and dissemination and increased host survival in the murine pneumonia model. This distinction is important because it indicates that the serum resistance and murine virulence phenotypes occur in different host contexts and should not be interpreted as evidence for the same mechanism. Specifically, because mouse serum exhibited minimal bactericidal activity against Kp38088 under the conditions tested, our data do not establish resistance to complement-mediated bactericidal activity as the basis for the attenuated virulence of the ΔGM000287 mutant in mice. Thus, the contribution of GM000287 to bacterial fitness during murine infection cannot be explained solely by resistance to serum bactericidal activity. The mechanism underlying this *in vivo* phenotype remains to be determined.

In summary, we identify GM000287 as a conserved T2SS-associated envelope factor that promotes resistance to human complement-mediated killing and contributes to *Kp* fitness and virulence during murine infection. Loss of GM000287 increases C3b production and factor B deposition during human serum exposure, consistent with enhanced alternative pathway amplification. Together, these findings establish GM000287 as a conserved determinant of *K. pneumoniae* fitness across distinct host-associated environments.

## Methods and materials

### Ethics statement

All animal experiment protocols and procedures were approved by the Institutional Animal Care and Use Committee (IACUC) of Shenzhen People’s Hospital, the First Affiliated Hospital of Southern University of Science and Technology. Approved animal protocol number: AUP-230531-ZK-0195-01.

### Bacterial strains

Clinical *Klebsiella pneumoniae* isolate Kp38088 was originally recovered from blood cultures of a patient at the First Affiliated Hospital of Zhejiang University, Hangzhou, China. Kp38088 is an ST11-KL64 hypervirulent CRKP strain. Additional clinical isolates, including Kp26529 (ST11-KL47, hypervirulent CRKP) and Kp39468 (ST11-KL64, hypervirulent CRKP), were also obtained from the same hospital [9]. The reference CRKP strain *K. pneumoniae* ATCC 700603 was purchased from the American Type Culture Collection (ATCC). *K. pneumoniae* ATCC 43816 (KPPR1; ST493-K2) is a well-established classical strain widely used in murine acute pneumonia models and was obtained from ATCC. The hypervirulent *K. pneumoniae* strain NTUH-K2044 (ST23-K1, O1) was originally isolated in Taiwan [52] and is maintained in our laboratory. Detailed information was included in S14 Appendix Dataset sheet 5.

Antibiotic susceptibility testing was performed to identify effective antibiotics which was used for genetic engineering or cell experiments, including 20μg/ml chloramphenicol, 30μg/ml tetracycline, 50μg/ml kanamycin, 50μg/ml spectinomycin, 25μg/ml G418, 50μg/ml dihydrostreptomycin, 50μg/ml apramycin, 200μg/ml hygromycin B, 4μg/ml polymyxin B, 15μg/ml trimethoprim lactate and 200μg/ml roxithromycin. Kp38088 strain was sensitive to 200μg/ml Hygromycin B, and 50μg/ml apramycin treatment.

A CRISPR-Cas9-mediated genome-editing method was used to generate mutants [53]. Kanamycin resistance gene at the plasmid of pSGKP-Km was replaced by hygromycin B (hph) and termed as pSGHph. The primers used for mutant construction and the strains used in this study are listed in S14 Appendix Dataset sheet 5.

The bacteria were grown at LB agar plates and in LB Medium with shaking at 37°C. Optical density 600 (OD_600_) was measured by nanodrop (Thermo Scientific, USA) with a 10mm path length cuvette, and the cultures were diluted in 1 × PBS as indicated CFU in the figures for each experiment.

For complementation studies, the plasmid pACYC184 was modified by replacing the tetracycline resistance cassette with a hygromycin B resistance cassette, generating the vector p184-hph. To complement the GM000287 deletion mutant, approximately 250 bp upstream of the GM000287 coding sequence, together with the full-length GM000287 open reading frame fused with a C-terminal 3 × Flag tag, was amplified and cloned into p184-hph, resulting in the constitutive expression plasmid P0287. To generate a lower-expression complementation construct, the native upstream regulatory region was replaced with the synthetic constitutive promoter J23103 and ribosome binding site B0033, generating plasmid J0287. The sequences of the J23103 promoter and B0033 ribosome binding site were obtained from the iGEM. The complementation plasmids were introduced into the corresponding mutant strains by electroporation. Transformants were selected and maintained on agar plates or in liquid medium supplemented with 200 μg/mL hygromycin B to ensure plasmid retention.

### Cells and *Kp* infection

Mouse lung epithelial cell (MLE-12;CRL-2110, ATCC) were cultured in Dulbecco’s Modified Eagle Medium (DMEM) supplemented with high-glucose, 10% heat-inactivated fetal bovine serum (FBS) and gentamicin (10μg/ml).

Mouse Bone-marrow-derived macrophages (BMDMs) were cultured in DMEM supplemented with 20ng/ml Macrophage colony-stimulating factor (M-CSF), 10% heat-inactivated fetal bovine serum, gentamicin (50μg/ml) and 10 mM L-glutamine. BMDM were maintained in 5% CO_2_ at 37°C. Half of cell culture media were gently removed and replaced with the same volume with 20ng/ml M-CSF and 10μg/ml gentamicin on day 3 and 6. On day 7, the adherent BMDM were digested with trypsin and seeded into 24-well plates for infection assay.

For adhesion assays, 5 × 10^5^ cells of MLE-12 or 5 × 10^6^ cells of BMDM were seeded into 24-well plates and infected with bacteria at a multiplicity of infection (MOI) of 10, 25 and 50 respectively. After one hour incubation at 37°C in 5% CO_2_, the monolayers were washed three times with 1 × PBS to remove the non- adherent bacteria. Cells were then released with 0.5% Triton X-100 and serial dilutions were plated on LB agar plates for CFU enumeration.

For epithelial cells internalization assays and macrophages intracellular killing assays, cells were infected at a MOI of 10, 25 and 50 and incubated for one hour at 37°C in 5% CO_2,_ then washed three times with 1 × PBS. DMEM culture medium containing apramycin (200 μg/ml) was added for additional culture time as indicated in the figure. Cells were lysed and intracellular bacteria were quantified as described above.

### Mouse lung infection model of Kp38088

Six- to eight-week-old female C57BL/6J mice purchased from GemPharmatech were used for mouse lung infection model. Mice were anesthetized by intraperitoneal injection of 0.20 ml 2.5% v/v 2,2,2-tribromoethanol (Avertin). A 22-G catheters (BD) were inserted into mice trachea through the mouth and 40 μl of the diluted inocula was intratracheally instilled by mice. Mice were held vertically for 10 seconds after the inoculation and were monitored for 7 days for survival experiments. To determine the bacterial burden in the lung, spleen and liver, infected mice were euthanized at day 2, and 4 post infection, the whole organs were harvested and homogenized in 1ml 1 × PBS. The mixture of homogenized samples was plated on LB agar plates for enumeration of colony forming units (CFU). All mouse experiments were approved by the Institutional Animal Care and Use Committee (IACUC) of Shenzhen People’s Hospital, the First Affiliated Hospital of Southern University of Science and Technology.

### Histopathology

C57BL/6J mice were infected with 2.0 LD_50_ Kp38088 and mutant strains. Mice were euthanized at 2 dpi and tissues were collected at indicated time points and were fixed in 10% neutral buffered formalin. Tissues were placed in cassettes and processed with a Donatello series 3 (Diapath, Italy) on a 12-hour automated schedule (Wuhan Junjie Electronics CO., Ltd) using a graded series of ethanol, xylene, and Paraplast Extra. Embedded tissues were sectioned at 5 μm with LEICA RM2016 microtome (Leica, German) and dried overnight at 42°C prior to staining with hematoxylin and eosin (H&E). Images were obtained using a Nikon ECLIPSE Ti2-U microscope.

### Growth curve analysis

2 μl of overnight cultures were diluted in 200 μl of LB at 96-well plates and incubated at 37°C with continuous, normal shaking in a BioTek Synergy H1 Analyzer (Bio Tek Instruments, USA), OD_600_ was measured and recorded every 30 minutes.

### Serum bactericidal assay

Bacterial survival in serum was performed according to previously described protocols with minor modifications [54]. Briefly, exponential-phase Kp38088 were washed and resuspended in 1 × PBS. 25 μl of 1 × 10^4^ or 1 × 10^6^ CFU bacteria in 1 × PBS were added into human serum (OriBiotech) for a total volume of 100 μl, the concentration of human serum in the mixture ranges from 25% to 75% as indicated in the figure. Serum was diluted in 1 × PBS prior to addition of bacteria. The mixture was incubated in 96-well plate at 37°C with shaking in a BioTek Synergy H1 Analyzer (Bio Tek Instruments, USA). CFU of the mixture was enumerated at indicated time points and the efficacy of serum bactericidal activity was calculated.

### ELISA

The bacteria whole-cell ELISA was performed as previously reported [55]. Briefly, the exponential phase of different Kp38088 strains complement with P0287 were resuspended in 1 ml sterile 1 × PBS to an OD_600_ = 0.3. Bacteria were fix with 4% (w/v) paraformaldehyde, followed by three times 1 × PBS washing, and then resuspended in coating buffer (100 mM bicarbonate/carbonate buffer, pH 9.6) to a final OD_600_ as indicated in the figure. 100 μl of this suspension were added into the wells of 96-well ELISA plates (Beyotime, PA068S) and incubated overnight at 4°C. The wells were then washed three times and blocked with blocking buffer (5% dried milk in 1 × PBST). 100 μl of primary antibody (mouse anti-Flag, rabbit anti-RNAP or BamA) diluted at 1:2500 and secondary HRP conjugated goat anti-mouse IgG or goat anti-rabbit IgG antibody (Huabio, M1403-2) in blocking buffer diluted at 1:10000 was subsequently added and incubated for one hour at 25°C. 100 μl of 3,3’,5,5’-Tetramethylbenzidine (TMB) substrate and stop solution was subsequently added, absorbance values were measured at 450 nm with wavelength correction of 570 nm using a microplate reader (Synergy H1, BioTek).

To evaluate the interaction of T2SS and GM000287 with complement components, 3 × 10^7^ CFU of *Kp* were incubated with final concentration of 80% normal human serum in a 96-well plate for a total volume of 150 μl at 37°C for 40 minutes with shaking. Following centrifugation, the supernatant were analyzed by ELISA to quantify C3b according to the manufacturer’s instructions.

### Flow cytometry analysis

Bacterial binding of Cfb (Bb fragment) was determined as previously described [16]. Briefly, 3 × 10^7^ CFU of bacterial strains were resuspended in 30 μl 1 × PBS and incubated with 120 μl normal human serum in a 96-well plate for 45 min at 37°C with gentle agitation. After incubation, bacteria were washed once with 1 × PBS containing 0.5% BSA and fixed in 4% paraformaldehyde in 1 × PBS for 30 min at room temperature. Fixed bacteria were then washed twice with 1 × PBS containing 0.5% BSA and incubated with a PE-conjugated mouse anti -human Cfb (Bb fragment) antibody (1:100 dilution) in 100 μl 1 × PBS containing 0.5% BSA for 30 min at 4°C. After incubation, bacteria were washed twice with 1 × PBS containing 0.5% BSA and resuspended in 500 μl 1 × PBS, cells were processed with the Becton Dickinson (BD) LSRII flow cytometer and All flow cytometry data were analyzed with FlowJo v10 software.

To characterize immune cell infiltration in the BALF, mice were intratracheally infected with 2.0 LD_50_ of Kp38088 or the indicated mutant strains. At 1 or 2dpi, mice were euthanized and BALF was collected for total leukocyte enumeration and flow cytometric analysis. BALF cells were treated with ammonium-chloride-potassium (ACK) lysis buffer to remove erythrocytes, and the lysis reaction was terminated by the addition of 10% fetal bovine serum (FBS) in 1 × PBS. Cells were then washed and resuspended in PBS. Total white blood cell (WBC) numbers in BALF were determined using an improved Neubauer hemocytometer.

For flow cytometric analysis, BALF cells were resuspended in FACS staining buffer and incubated with anti-mouse CD16/32 antibody (Fc block) for 15 min at 4°C to minimize nonspecific Fc receptor-mediated antibody binding. Cells were subsequently stained with a cocktail of fluorochrome-conjugated antibodies against the indicated surface markers, including CD45 (PE), CD11b (APC), Ly6C (APC-Cy7), and Ly6G (BV605). All antibodies were purchased from BioLegend. Stained cells were washed, resuspended in FACS staining buffer, and analyzed by flow cytometry as described above.

### Mass spectrometric analysis of *Kp* 38088 culture supernatants

WT and Δ*gspDE* strains were grown to the mid-exponential phase in LB broth (OD600 = 0.7 ~ 1.0), H_2_O_2_ was added to a final concentration of 3 mM, and cultures were further incubated for 1.5 h to induce oxidative stress. An equal volume of 1 × PBS was added to parallel cultures as the negative control. Subsequently, 100 ml of 0.22 μm-filtered culture supernatant was collected by centrifuge and protease inhibitor cocktails was added, sodium deoxycholate (DOC) was then added which yielded a final volume concentration of 0.03%. The mixture was incubated for 20 min at room temperature, followed by adding trichloroacetic acid (TCA) with the final concentration of 10%. The mixture was incubated on ice for 12 hours with gentle mixing. Subsequently, the cell pellets were collected by centrifugation at 13,000 × g for 15 min at 4°C. The TCA and DOC were removed by washing with cold-acetone. Finally, the pellets were resuspended in 1 ml of 10 mM Tris-HCl (pH 7.5) with 2% SDS and protease inhibitors. Samples were further analyzed by mass spectrometry (Shenzhen Winovate Bio-Techonology).

### Western blotting

Cell culture supernatant was precipitated using TCA as above mentioned, and the whole cell proteins were collected directly from bacteria pellets. Samples were electrophoresis and transferred onto PVDF membrane followed by 5 × loading buffer added and heated. Membranes were subsequently blocked and probed with primary antibodies anti- Flag,anti-RNAP, anti-MalE and anti-BamA at 1:5000 ~ 10000 for the whole protein detection and at 1:1000 for supernatant protein detection (Cusbio Technology). HRP-conjugated Goat anti- mouse or anti-rabbit secondary antibodies diluted at 1:20000 were used for detection. Images were acquired by the Mini Chemiluminescent Imaging System.

### Reverse transcription quantitative PCR (qRT-PCR)

Bacteria pellets were incubated with 10 mg/mL lysozyme prior to the addition of RL lysis buffer. Total RNA was purified according to the manufacturer’s instruction (Cwbio). Reverse transcription was performed using the Evo M-MLV RT Mix Kit (Accurate Biology). Quantitative real-time PCR was performed using the SYBR Green Premix Pro Taq HS qPCR Kit (Accurate Biology) on an Applied Biosystems qPCR System for cDNA amplification and quantification. The expression levels of bacterial genes were normalized to 30S ribosomal protein S12 (rpsL).

### UID total RNA-seq

The integrity of the RNA was verified using the LabChip GX Touch system (Revvity) and RNA concentration was then determined using a Qubit 3.0 fluorometer with the RNA broad range assay kit (Thermo Fisher, Q10210). The RQN/RIN ratio and quantity of RNA is higher than 4.0 and 500ng respectively. For library preparation, rRNA was depleted firstly (Vazyme, China, N406 for human/mouse/rat, N417 for bacteria), and then components of KCTM Digital Stranded mRNA library prep kit (Wuhan Seqhealth Co., Ltd. China) were used for stranded total RNA sequencing library preparation following the manufacturer’s instruction. The kit eliminated duplication bias and errors in PCR and sequencing steps by labelling the pre-amplified cDNA molecules with unique molecular identifier (UID) of 12 random bases. The library preparation included the enrichment of PCR products corresponding to fragments ranging from 200 to 500 base pairs. The enriched libraries were quantified and sequenced using the PE150 sequencing mode to generate paired-end reads (Illumina).

### Quantification of capsule production

Capsule production was quantified by measuring uronic acid content as previously described [56]. Briefly, 500 μl of log-phase bacterial cultures were normalized to equivalent CFU and mixed with 100 μl of 1% Zwittergent 3–14 detergent prepared in 100 mM citric acid. Samples were incubated at 56°C for 20 min to extract capsular polysaccharides. After centrifugation at 14,000 × g for 2 min, 300 μl of the supernatant was collected and mixed with 1.2 mL of ice-cold absolute ethanol, followed by incubation at 4°C for 20 min to precipitate the polysaccharides.

The precipitated material was collected by centrifugation at 14,000 × g for 5 min, air-dried, and washed with 70% ethanol. The pellet was subsequently dissolved by incubation at 56°C. Then, 1.2 mL of 12.5 mM sodium tetraborate in concentrated sulfuric acid was added, and samples were incubated at 100°C for 5 min. After cooling on ice for 10 min, 20 μl of 0.15% 3-phenylphenol prepared in 0.5% NaOH was added, followed by a 5-min incubation at room temperature. Finally, 200 μl of each reaction mixture was transferred into a 96-well microplate, and absorbance was measured at 520 nm.

### Bioinformatic identification of GM000287 and T2SS components

The bacterial genomes used in this study are listed in S12 Appendix Dataset sheet 3, and the corresponding genome sequences were downloaded from the National Center for Biotechnology Information (NCBI) database. Homologs of GM000287 and T2SS components were identified using the easy-search module of MMseqs2 (version 13.45111). The GM000287 protein and T2SS components from *Klebsiella pneumoniae* Kp38088 were used as query sequences. Searches were performed using the default MMseqs2 settings, and hits with alignments covering >50% of the corresponding query protein were retained for subsequent analysis.

A total of 1,524 representative bacterial genomes and 326 *Klebsiella* genomes were included in the GM000287 homology analysis [57,58].

An additional set of 190 representative Gammaproteobacteria genomes was used for comparative analysis of GM000287 and T2SS components. Fourteen core T2SS proteins from Kp38088 were used as query sequences for MMseqs2 searches using the criteria described above. Genomes containing homologs of at least one of the 14 T2SS core proteins were designated as T2SS-positive. The presence or absence of GM000287 and the 14 T2SS components was recorded for each genome and used for subsequent comparative analyses.

**Significance**

*Klebsiella pneumoniae* causes life-threatening invasive infections and poses a major global health threat. While the polysaccharide capsule is a well-known mediator of serum resistance, capsule-independent mechanisms that enable complement evasion remain incompletely understood. Here, we identify GM000287 as a previously uncharacterized and highly conserved determinant of human serum resistance and *in vivo* virulence. Loss of GM000287 increases C3b production and factor B deposition during human serum exposure, consistent with enhanced alternative pathway amplification. In contrast, the virulence phenotype of the GM000287 mutant in mice, in which serum bactericidal activity against Kp38088 is minimal, indicates an additional role for GM000287 during infection. These findings identify GM000287 as a conserved bacterial factor with distinct contributions to human serum resistance and bacterial fitness during infection.

## Supporting information

S1 Fig Survival curves of WT- and ΔpulA-infected mice. C57BL/6J mice (n = 12) were intratracheally infected with 2.0 LD_50_ of WT and Δ*pulA* strains. Survival was monitored for up to 7 dpi. Statistical significance of survival rate was determined using Mantel-cox test.
(TIF)

S2 Fig (A) Gate strategy of flow cytometry. The live cell populations were gated as CD45^+^CD11b^+^Ly6C^+^ for monocyte cell and CD45^+^CD11b^+^Ly6G^+^ for neutrophil cell. (B) Total white blood cells from C57BL6/J mice BALF at 1 and 2 dpi infected with 2.0LD_50_ of WT and Δ*gspDE* strains.
(TIF)

S3 Fig Effects of GM000287 deletion and complementation on bacterial growth in vitro. The growth kinetics of WT, Δ0287, and complemented strains (Δ0287*::*P0287 and Δ0287*::*J0287) were comparable during growth in LB medium. WT, Δ0287, and Δ0287*::*J0287 exhibited comparable growth kinetics, indicating that deletion of GM000287 or complementation with J0287 did not significantly affect bacterial growth. In contrast, Δ0287*::*P0287 showed a growth defect compared with Δ0287*::*J0287, which may be attributed to excessive expression of GM000287. Statistical significance was determined using two-way ANOVA test ***, P < 0.001; **, P < 0.01; *, P < 0.05.
(TIF)

S4 Fig GM000287 expression during bacterial growth. GM000287 transcript levels were quantified by qRT-PCR in WT and Δ*gspDE* during exponential and stationary phases. Data were represented the means ± SEM from two independent experiments. Statistical significance was determined using two-way ANOVA. **, P < 0.01; ns, not significant.
(TIF)

S5 Fig (A) Conservation analysis of T2SS locus among Gammaproteobacteria family. The prevalence of T2SS locus was calculated as the proportion of T2SS-positive strains within each species. (B) Genomic organization of the T2SS gene cluster and the location of GM000287 in the Kp38088 genome.
(TIF)

S6 Fig RNA-seq Analysis of WT vs Δ0287 (A) and WT vs. Δ0287::P0287 (B). Volcano plot showing differentially expressed genes in WT vs Δ0287 and WT vs. Δ0287::P0287. A threshold of 4-fold change (P < 0.05) was applied. Light blue dots indicate downregulated genes, light red dots represent upregulated genes, and grey dots denote genes with no significant changes.
(TIF)

S7 Fig (A-B) Bacterial adherence assay. Bacterial adherence was measured following a one-hour incubation with WT, Δ*gspDE* and Δ0287 strains at MOIs of 10 and 50 in BMDM and Murine Lung Epithelial-12 (MLE-12) cells. BMDM (A) and MLE-12 cells (B) were washed three times with 1 × PBS to remove non-adherent bacteria. Adherent bacteria were then enumerated by plating serial dilutions. **(C-D) Infection of BMDM and MLE-12 cell.** BMDM and MLE-12 cells were infected with WT, Δ*gspDE* and Δ0287 strains at MOIs of 10 and 50. After a two-hour incubation, cells were washed three times with 1 × PBS, and then 200 µg/mL apramycin was added for two hours to eliminate extracellular bacteria. Infected BMDM cells were cultured for additional 4 and 36 hours (C), infected MLE-12 cells were cultured for additional 4 hours (D), CFUs were enumerated by plating serial dilutions.
(TIF)

S8 Fig Capsule quantification by uronic acid assay. Capsular polysaccharide levels in WT, Δ*gspDE*, ΔGM000287, and Δ*wcaJ* strains were quantified using a uronic acid assay. The capsule production of Δ*gspDE* and ΔGM000287 mutants was comparable to that of the WT strain, whereas the ΔwcaJ mutant, used as a capsule-deficient control, exhibited a significant reduction in capsular polysaccharide levels. Data were represented as the means ± SEM from two independent experiments. Statistical significance was determined using unpaired two-tailed Student’s t-test. ***, P < 0.001; ns, not significant.
(TIF)

S9 Fig Complement-associated gene expression in BALF during K. pneumoniae pulmonary infection. C57BL/6J mice were intratracheally infected with 2.0 LD_50_ of Kp38088 or administered an equivalent volume of 1 × PBS as a control (n = 6 per group). BALF was collected at 48 h post-infection, and the transcript levels of C1q, C2, C3, C4, and Cfb were quantified by qRT-PCR. Statistical significance was determined using the Mann–Whitney U test. **P < 0.01; ***P < 0.001; ns, not significant.
(TIF)

S10 Appendix Dataset sheet 1. The RNA-seq analysis of Kp38088 grown in LB versus supplemented with 25% normal human serum.
(XLSX)

S11 Appendix Dataset sheet 2. The detailed information of mass spectrometry analysis of supernatant proteins between WT and Δ*gspDE.*
(XLSX)

S12 Appendix Dataset sheet 3. Genome information of 326 Klebsiella genus used for phylogenetic analysis of GM000287. Genome information of 1524 strains represented Proteobacteria, Actinobacteria, Firmicutes, Bacteroidetes and other phylums. Genome information of 190 strains represented Proteobacteria used for phylogenetic analysis of T2SS and GM000287.
(XLSX)

S13 Appendix Dataset sheet 4. The total RNA-seq analysis of WT vs Δ0287 (A) and WT vs. Δ0287::P0287 (B).
(XLSX)

S14 Appendix Dataset sheet 5. Primers, plasmids and Strains were used in this study.
(XLSX)

S1 File Raw images. The original uncropped and unadjusted western blotting images.
(PDF)
