# Supplementary material for: GM000287 is a conserved determinant of serum resistance and virulence in Klebsiella pneumoniae
Source: PLoS Pathog. 2026 Sep 24;22(9):e1014619. doi: 10.1371/journal.ppat.1014619 (PMC13623161; doi:10.1371/journal.ppat.1014619)
Supplement: Supplementary file 1 [file ppat.1014619.s015.pdf]

Original gel image

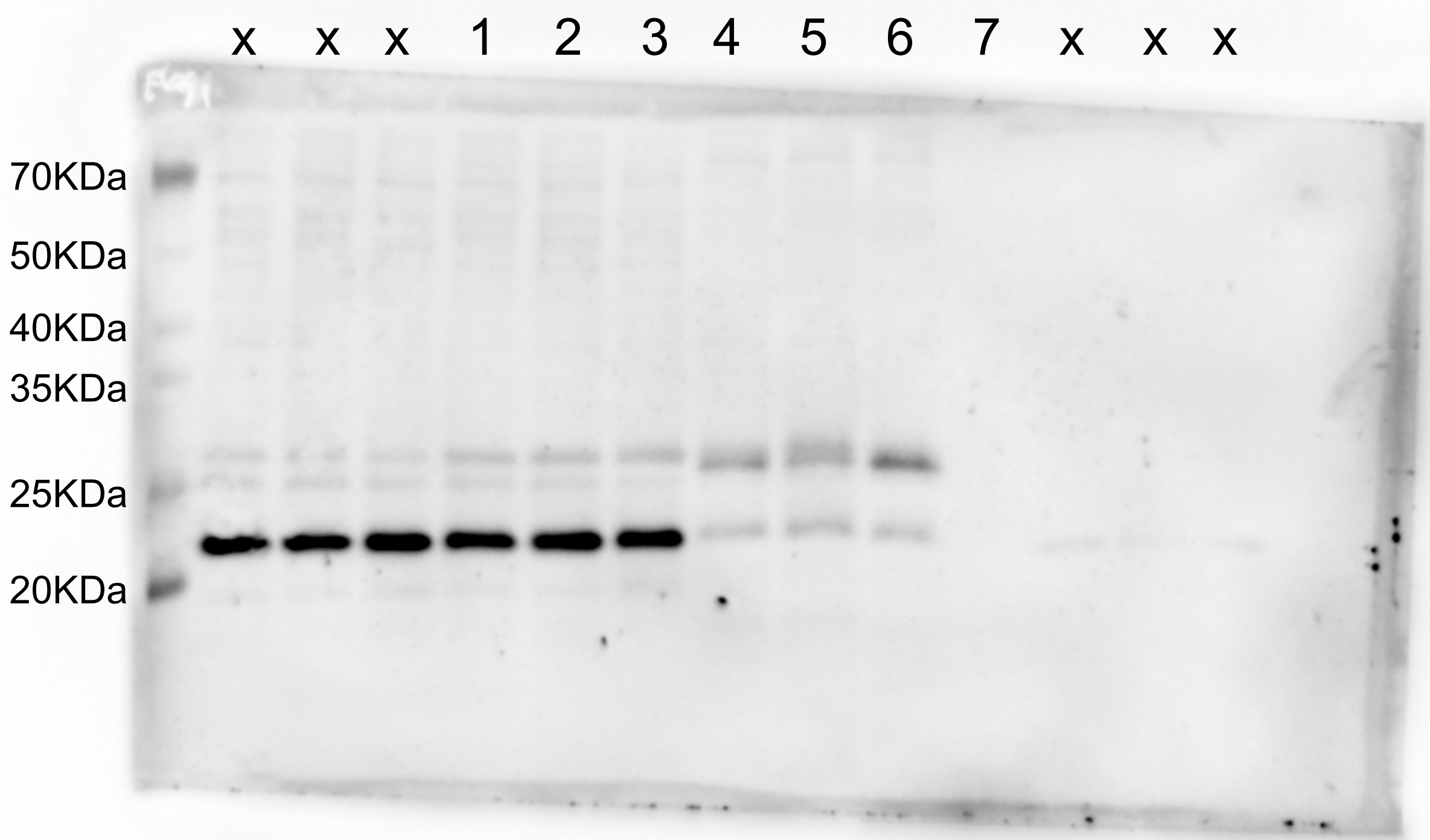

Lane 1: WT::P0287

Lane 2: Δ0287::P0287

Lane 3: ΔGspDE::P0287

Lane 4: WT::J0287

Lane 5: Δ0287::J0287

Lane 6: ΔGspDE::J0287

Lane 7: WT

Final gel image

Figure 4. F

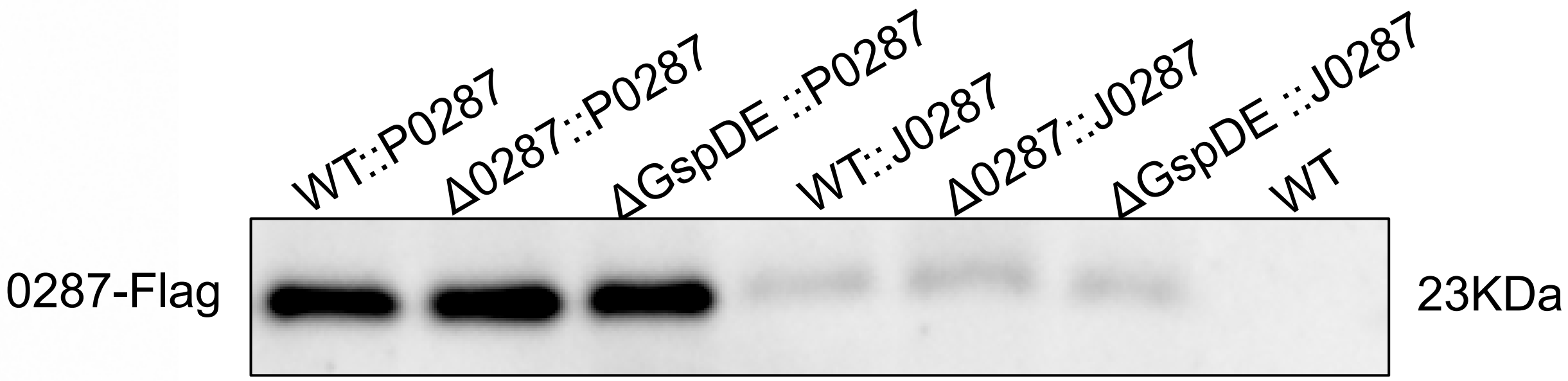

The samples were incubated with Flag-antibody overnight, and the image was captured with MinChemi610Plus imager.

Original gel image

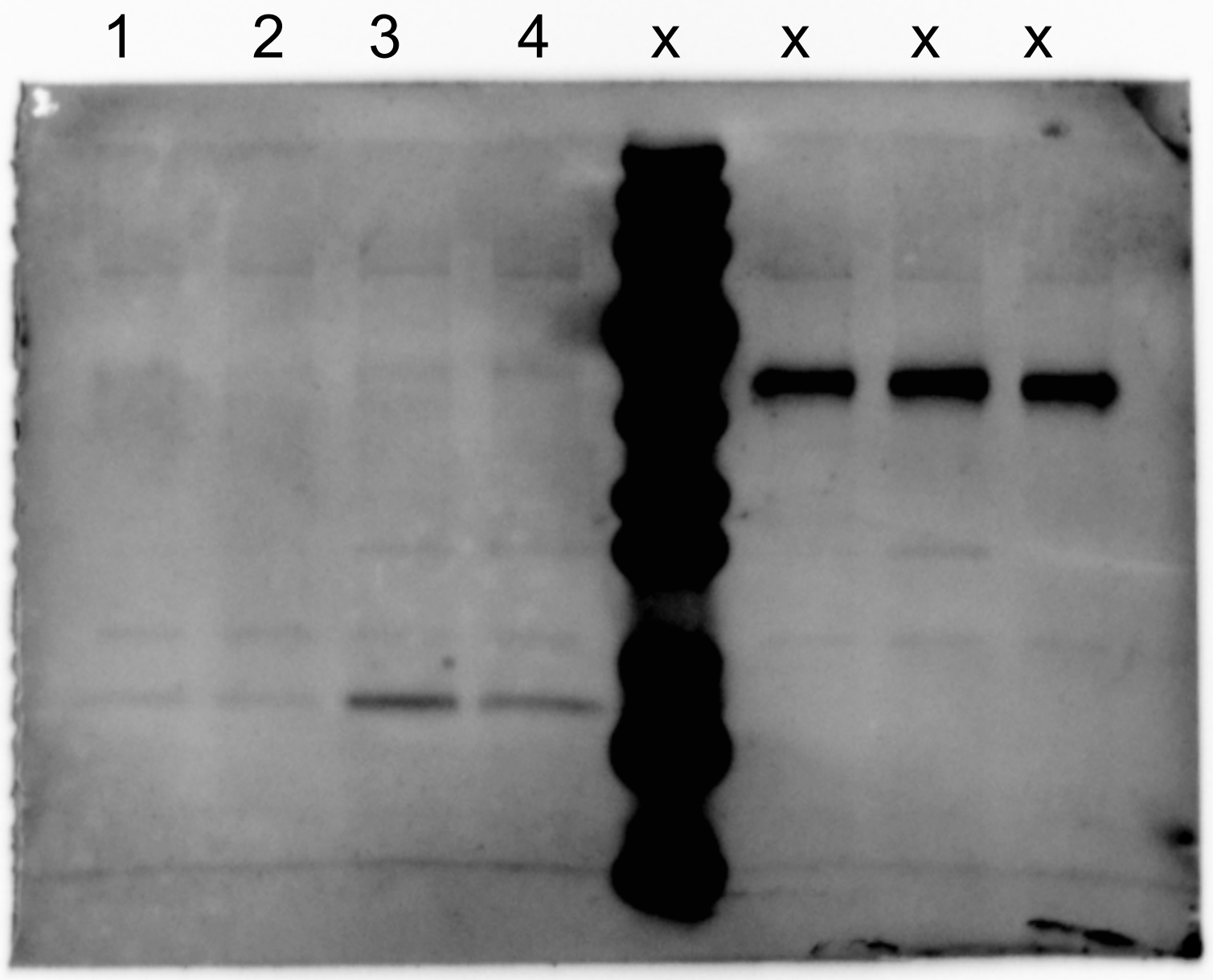

Marker image

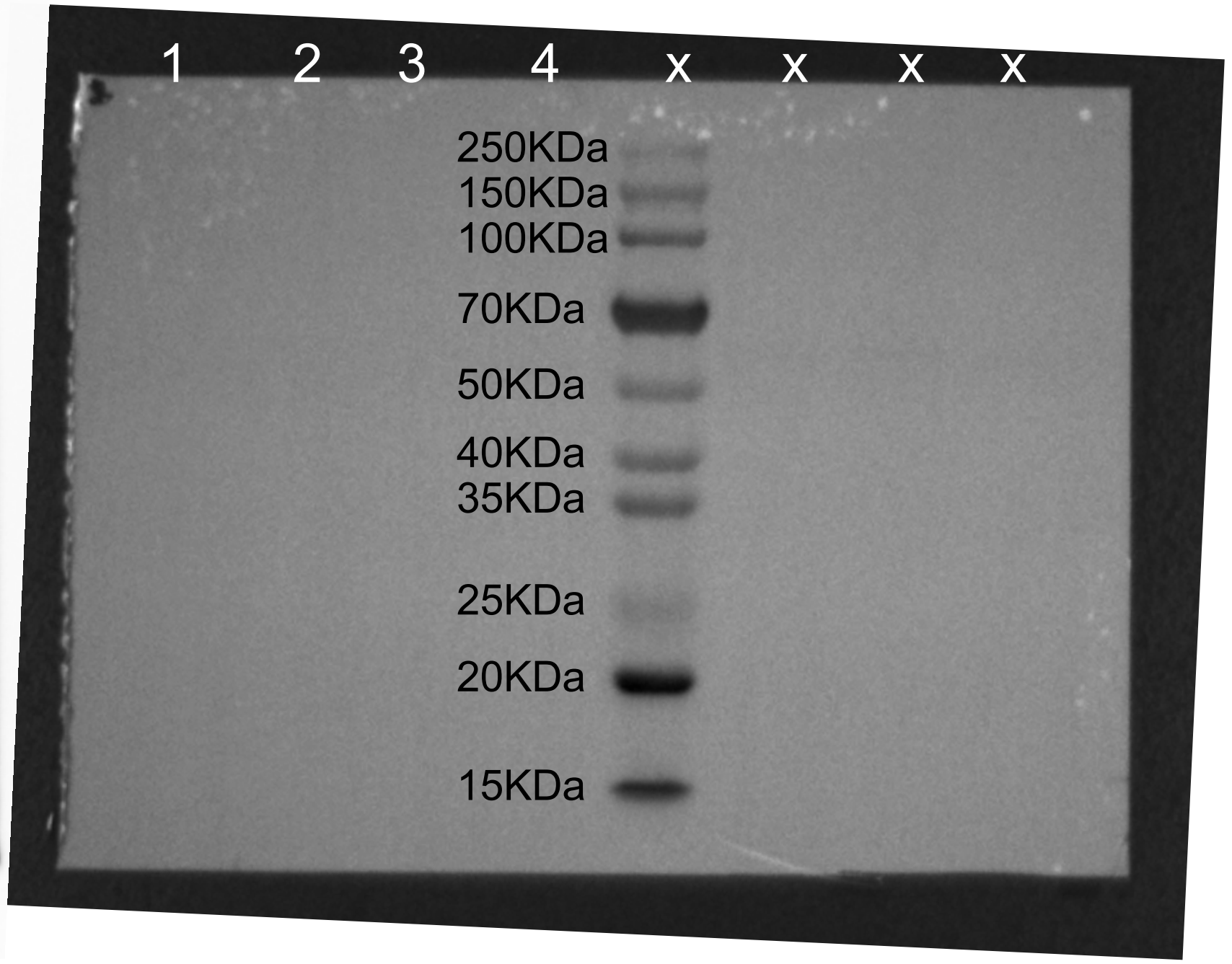

Final gel image

Figure 4. G

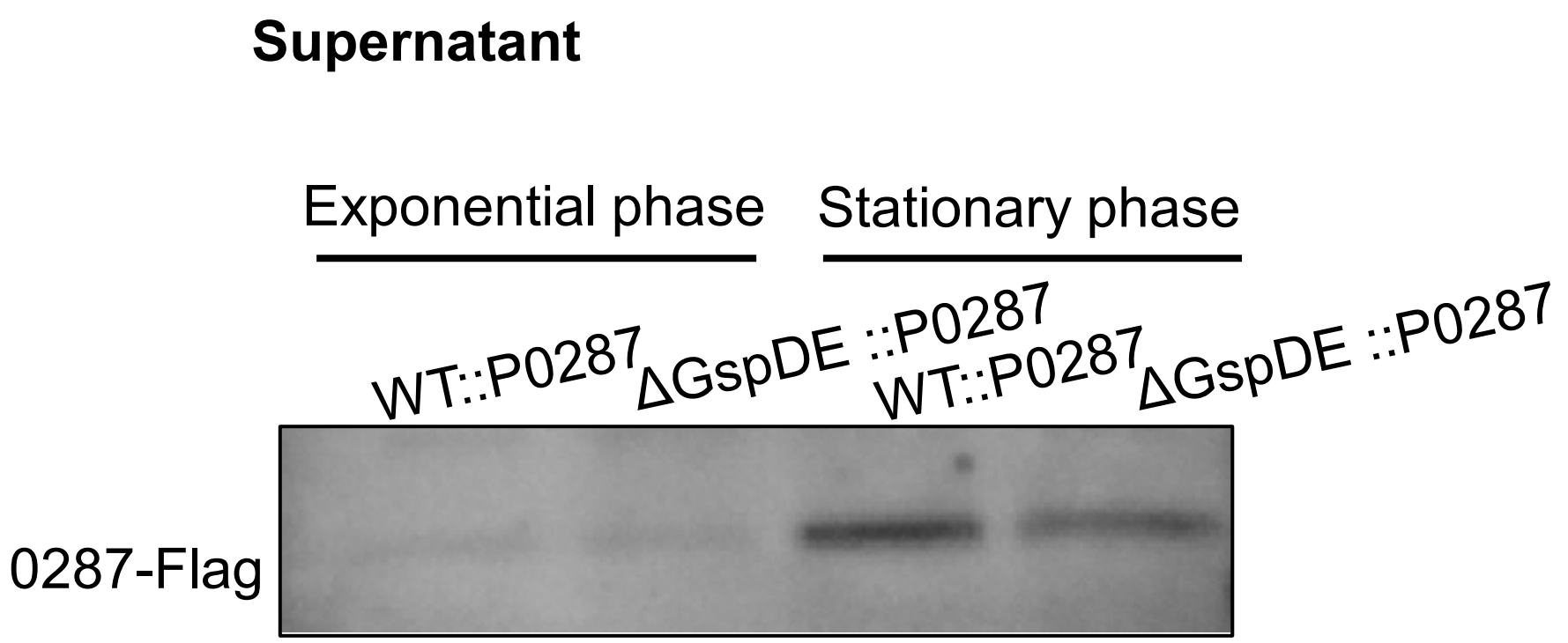

Lane 1: WT::P0287 Supernatant proteins collected at the exponential phase

Lane 2: ΔGspDE::P0287 Supernatant proteins collected at the exponential phase

Lane 3: WT::J0287 Supernatant proteins collected at the stationary phase

Lane 4: ΔGspDE::J0287 Supernatant proteins collected at the stationary phase

The samples were incubated with Flag-antibody overnight,

and the image was captured with MinChemi610Plus imager.

Origibal gel image

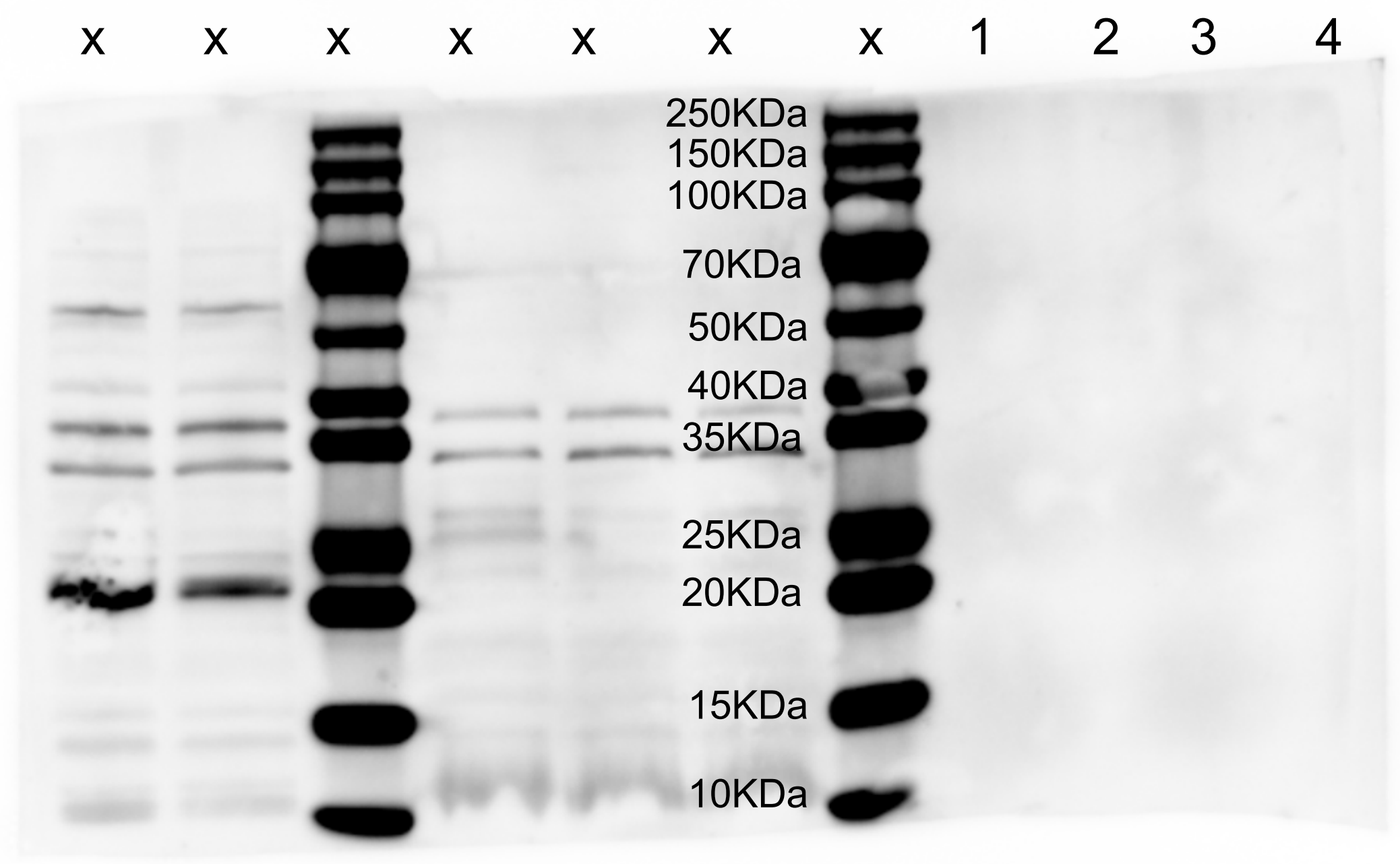

Lane 1: WT::P0287 Supernatant proteins collected at the exponential phase

Lane 2: ΔGspDE::P0287 Supernatant proteins collected at the exponential phase

Lane 3: WT::J0287 Supernatant proteins collected at the stationary phase

Lane 4: ΔGspDE::J0287 Supernatant proteins collected at the stationary phase

Final gel image

Figure 4. G

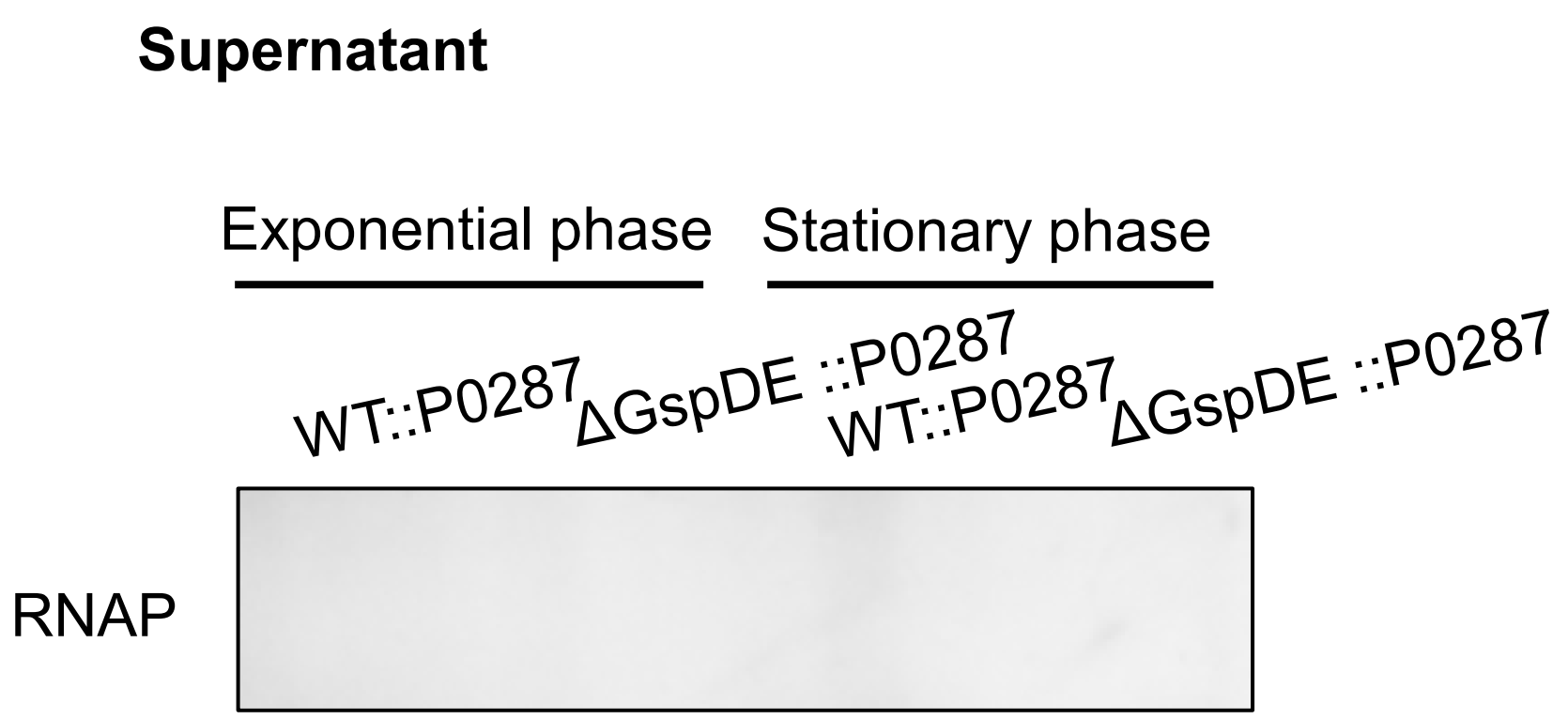

The samples were incubated with RNAP-antibody overnight,

and the image was captured with MinChemi610Plus imager.

Origibal gel image

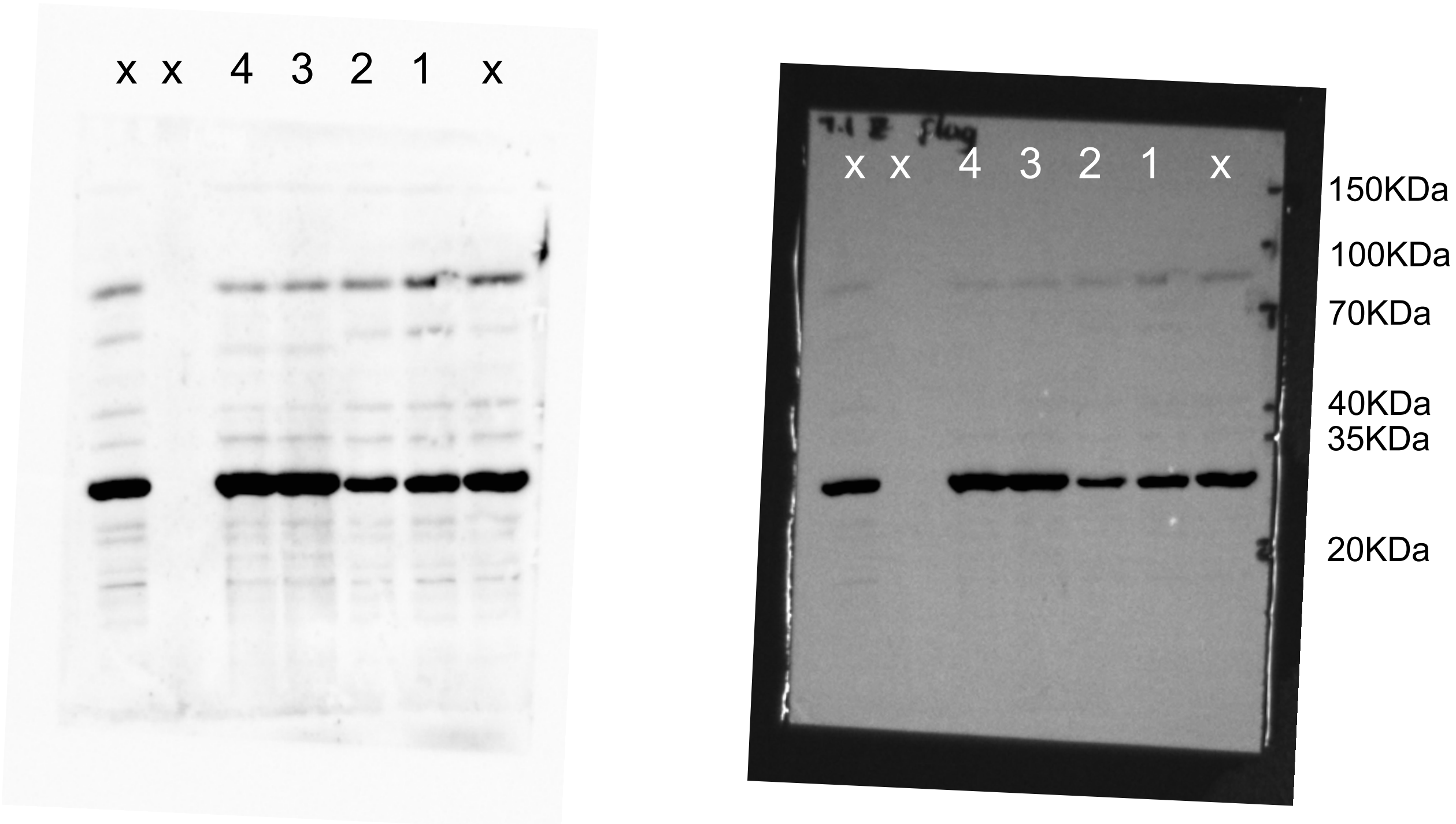

Lane 1: WT::P0287 Supernatant proteins collected at the exponential phase

Lane 2: ΔGspDE::P0287 Supernatant proteins collected at the exponential phase

Lane 3: WT::J0287 Supernatant proteins collected at the stationary phase

Lane 4: ΔGspDE::J0287 Supernatant proteins collected at the stationary phase

Final gel image

Figure 4. G

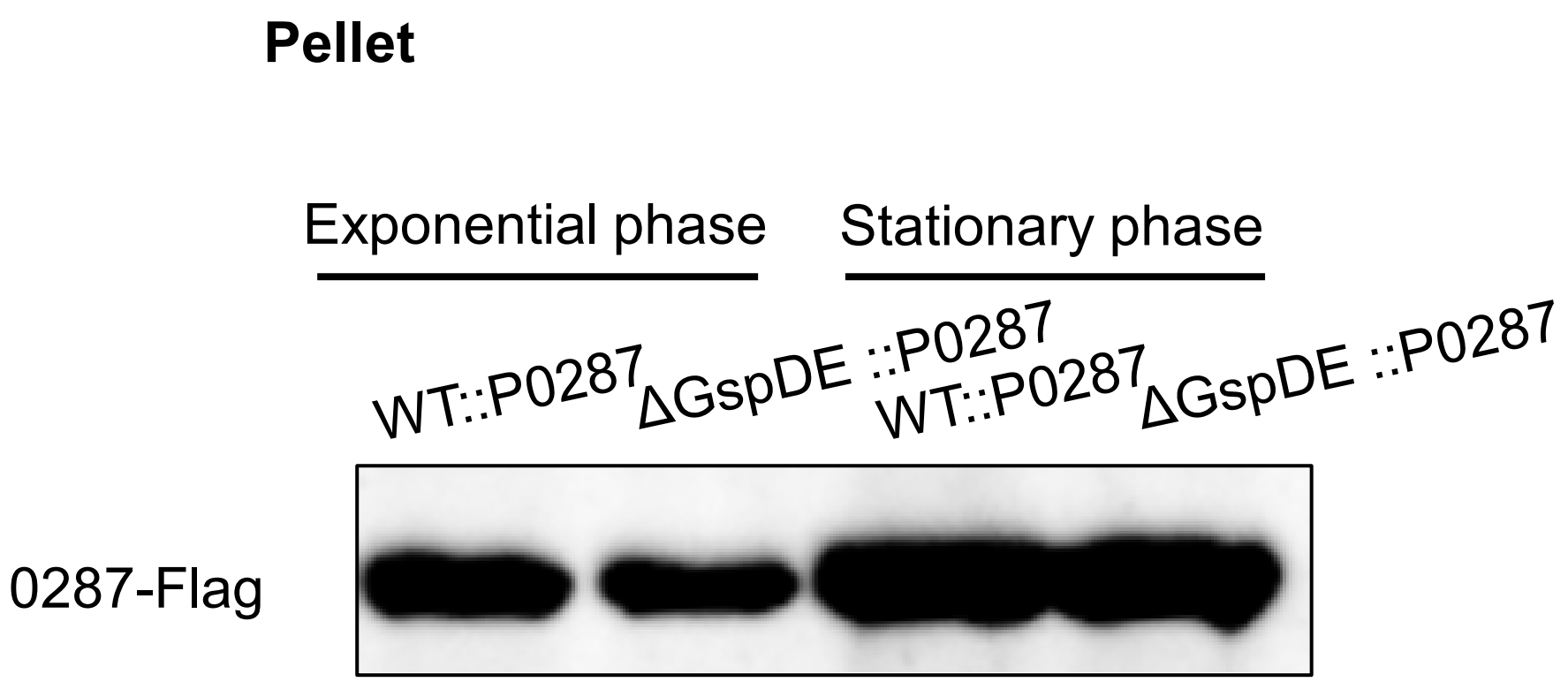

The samples were incubated with Flag-antibody overnight,

and the image was captured with MinChemi610Plus imager.

Origibal gel image

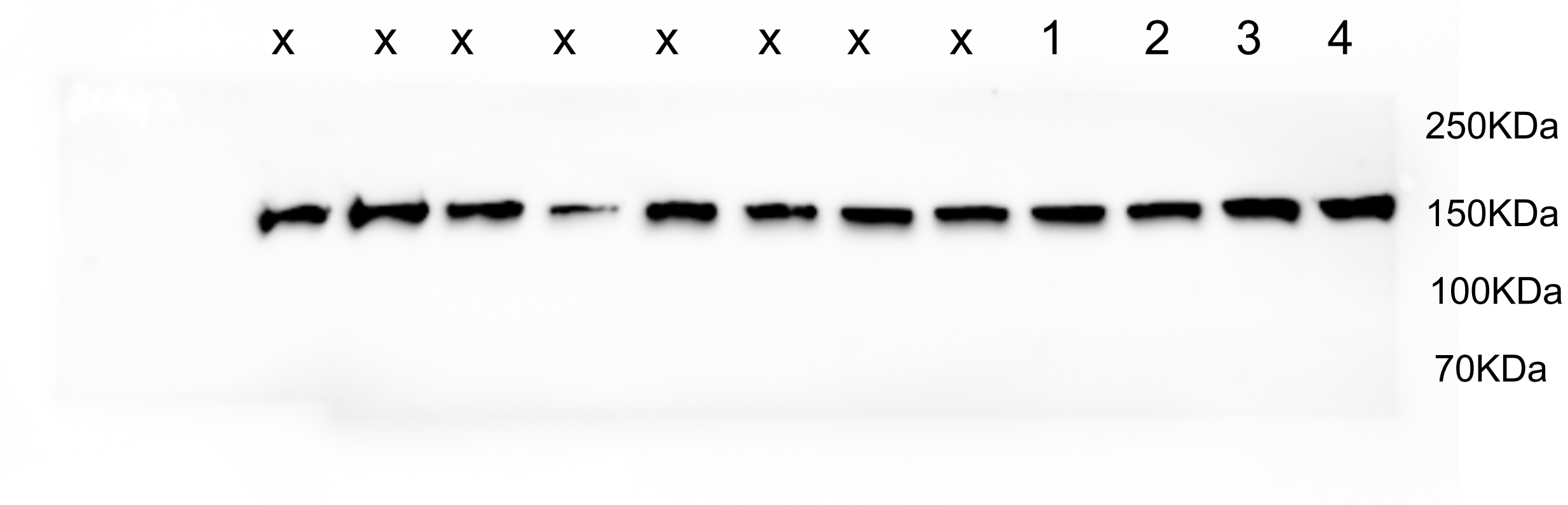

Lane 1: WT::P0287 Supernatant proteins collected at the exponential phase

Lane 2: ΔGspDE::P0287 Supernatant proteins collected at the exponential phase

Lane 3: WT::J0287 Supernatant proteins collected at the stationary phase

Lane 4: ΔGspDE::J0287 Supernatant proteins collected at the stationary phase

Final gel image

Figure 4. G

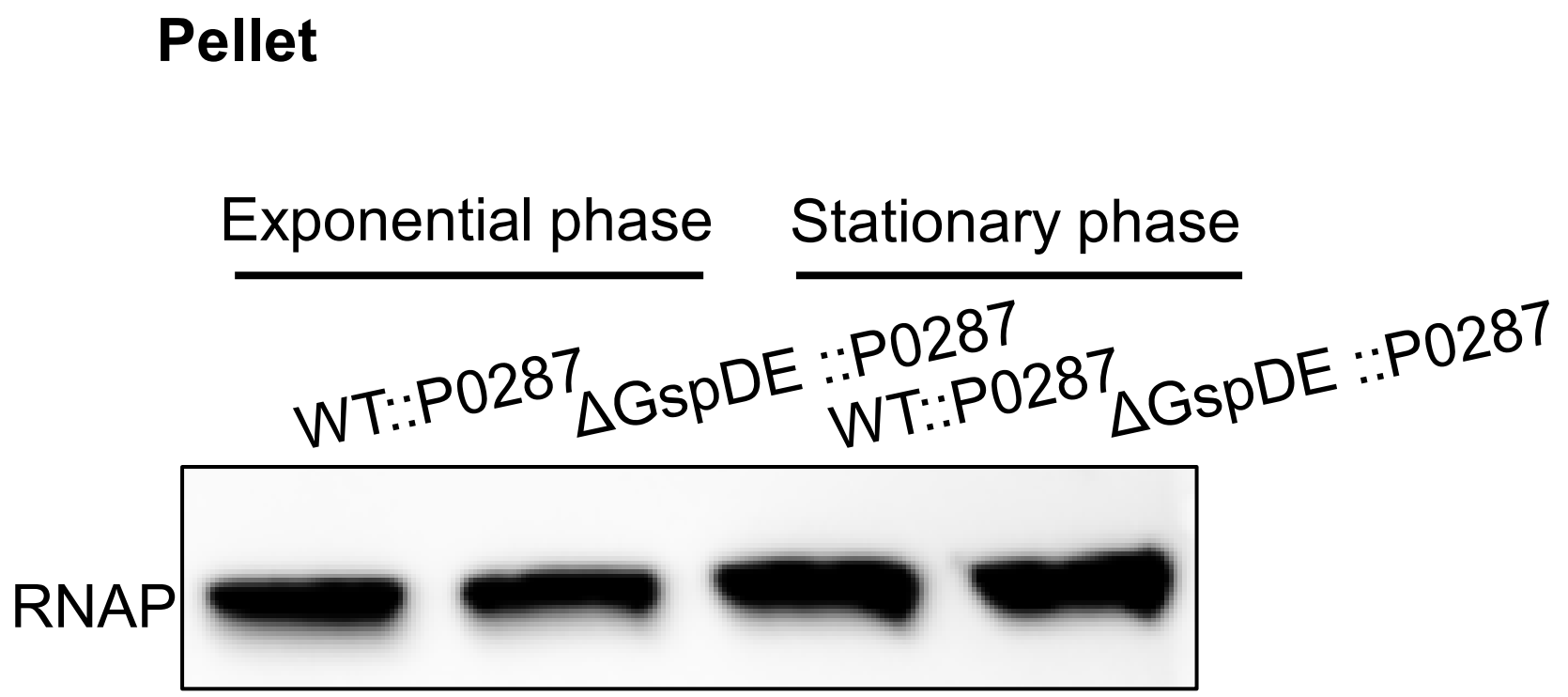

The samples were incubated with RNAP-antibody overnight,

and the image was captured with MinChemi610Plus imager.

Origibal gel image

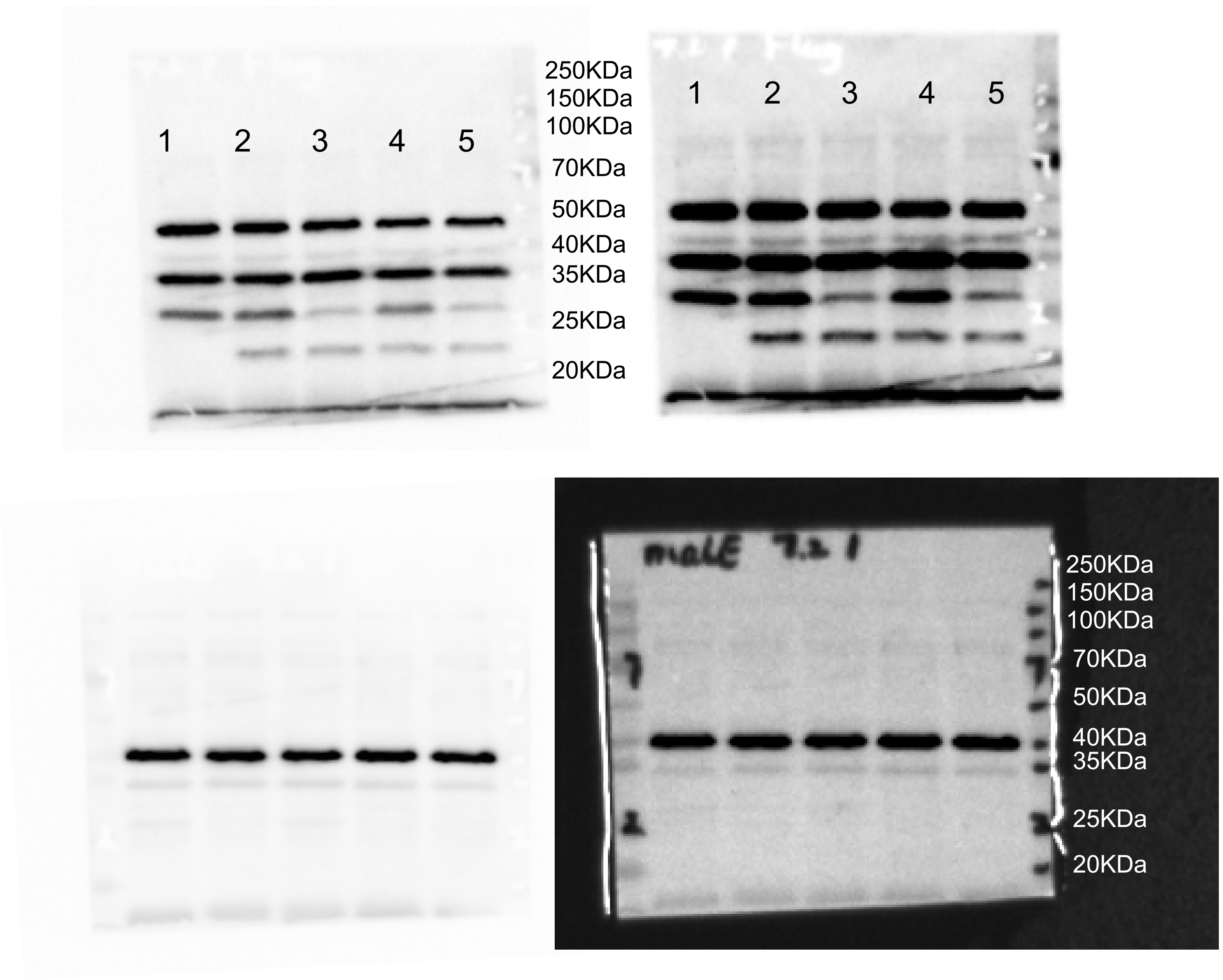

Final gel image

Figure 4. H

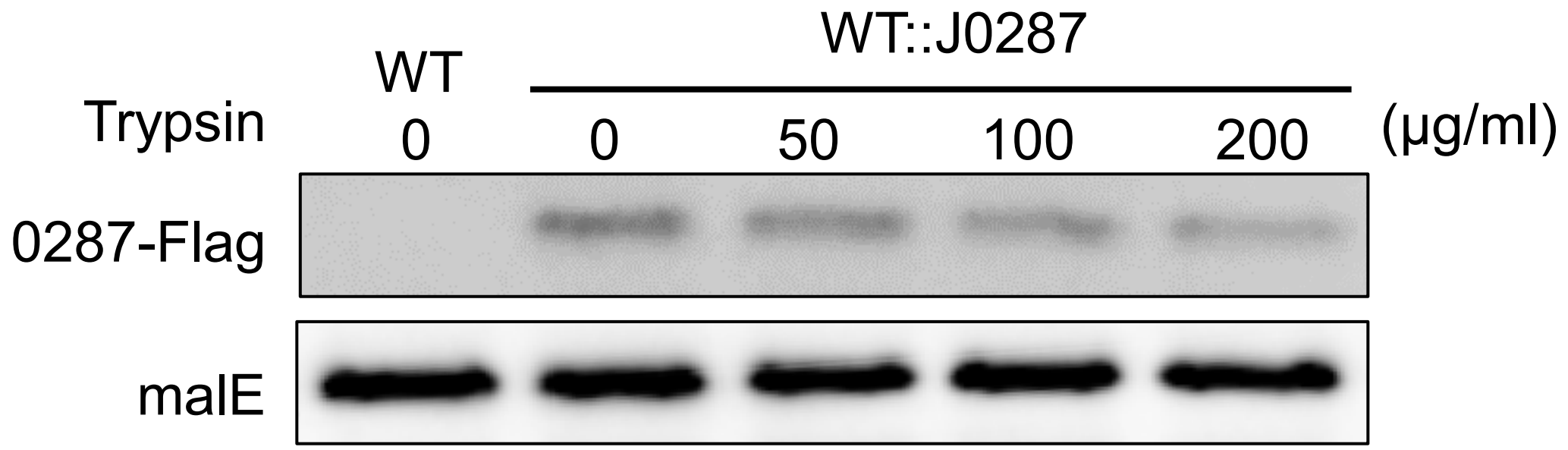

The samples were incubated with malE-antibody or Flag-antibody overnight,

and the image was captured with MinChemi610Plus imager.

Lane 1: WT

Lane 2: WT::J0287 with 0ug/ml trypsin incubation for 30min.

Lane 3: WT::J0287 with 50ug/ml trypsin incubation for 30min.

Lane 4: WT::J0287 with 100ug/ml trypsin incubation for 30min.

Lane 5: WT::J0287 with 200ug/ml trypsin incubation for 30min.
